# Americans’ Perspectives on Online Media Warning Labels

**DOI:** 10.3390/bs12030059

**Published:** 2022-02-23

**Authors:** Jeremy Straub, Matthew Spradling

**Affiliations:** 1 Department of Computer Science, North Dakota State University, Fargo, ND 58102, USA; 2 Department of Mathematics and Applied Sciences, University of Michigan Flint, Flint, MI 48502, USA; mjspra@umich.edu

**Keywords:** online media, warning labels, fake news, social media, deceptive content, age, gender, income, education, political affiliation

## Abstract

Americans are pervasively exposed to social media, news, and online content. Some of this content is designed to be deliberately deceptive and manipulative. However, it is interspersed amongst other content from friends and family, advertising, and legitimate news. Filtering content violates key societal values of freedom of expression and inquiry. Taking no action, though, leaves users at the mercy of individuals and groups who seek to use both single articles and complex patterns of content to manipulate how Americans consume, act, work, and even think. Warning labels, which do not block content but instead aid the user in making informed consumption decisions, have been proposed as a potential solution to this dilemma. Ideally, they would respect the autonomy of users to determine what media they consume while combating intentional deception and manipulation through its identification to the user. This paper considers the perception of Americans regarding the use of warning labels to alert users to potentially deceptive content. It presents the results of a population representative national study and analysis of perceptions in terms of key demographics.

## 1. Introduction

Jahng posited that “fake news” could be “the new social media crisis” [1] and Hopf cautioned that “fake science” was creating a potentially lethal “knowledge crisis” [2]. Sellnow, Parrish, and Semenas [3] note that even real crises and hoaxes have become difficult to disambiguate from fake ones. These are just some of the signs suggesting that online misinformation is reaching a level of global crisis. Concerns have been raised regarding election interference [4,5], and misinformation was even responsible for causing an armed standoff at a pizza parlor [6,7]. Significant misinformation has been spread concerning COVID-19 [8,9] and vaccination [10]. From the foregoing, it is clear that the power of social media to influence the population is demonstrable. The “fake news” problem joins a variety of other forms of misinformation and manipulation, such as propaganda, mystification, biased content, pseudoscience, clickbait, and conspiracy theories; however, while it may have some overlap with these, it has its own characteristics that arise from trying to present itself as a trusted news information source.

Kien [11] notes that, on social media, users’ “personal desires” are presented “as ‘truth’” in a significant departure from the concept of there being a “objective reality”. Instead, a “postmodern limbo” exists [11] under which, Koschorke [12] notes, tenants of academic thought models became “virulent under completely changed political circumstances”. This has resulted in notions of post-modernism being used to justify concepts like “post-truth” in ways never intended by the “liberal academic circles” they were developed in [12].

In response to these concerns, social media platforms, including Twitter [13], Facebook [14], and YouTube [15], have taken some steps towards transparency and protecting the public from deceptive online content. However, recent revelations concerning Facebook’s activities such as prioritizing profits over public safety [16], not being forthcoming with its own advisory board about its “cross-check” program [17], and not taking action upon discovering that the website “radicalized” users and created “fringe groups” [18] have raised public concern. Sicha [19] has even gone as far to suggest that social media could represent a “Tartarus … for humanity” (the term Tartarus comes from Greek mythology where it referred to the “lower” region of the “underworld, where the gods locked up their enemies” [20]) if left unchecked.

Some misinformation (or content that some consider to be misinformation) online is undoubtably due to legitimate mistakes, differences of opinion and non-malicious spreading. A variety of individuals and groups, however, have propagated misinformation via social media using bots and phony accounts with a specific purpose of misinforming the public to manipulate their opinions and actions [21]. Given the tremendous power that social media has been demonstrated to have over the public, gaining a better understanding of how negative outcomes associated with deceptive content can be avoided without impairing the basic principles of free speech, democracy, personal liberty, and free enterprise is paramount. To this end, this article presents and analyzes the results of a nationwide exploratory study in the United States regarding public perceptions regarding social media warning labels.

This paper continues with Section 2, which discusses prior work related to media labeling which this work builds upon. Section 3 presents the design of the national study and Section 4 presents the results from the national population representative study. Section 5, Section 6, Section 7, Section 8 and Section 9 analyze the data to identify differences in perception associated with age, education level, household income, political affiliation, and gender, respectively. Based on this, a comparative analysis of the public’s views is presented in Section 10, before the paper concludes in Section 11 and discusses needed areas of future work.

## 2. Background

This section describes previous studies which provides a foundation for the work presented herein. First, recent work on gauging the effectiveness of product labeling is discussed in Section 2.1. Next, prior studies on the identification and classification of fake news are presented in Section 2.2. Then, prior work related to the labeling of fake news and the sentiments surrounding it is discussed in Section 2.3. Finally, Section 2.4 presents prior work on labeling and fact checking.

### 2.1. Perceptions and Effectiveness of Product Labeling

Product and content labeling has a long history in the United States, dating back to the late 1890s and early 1900s. Early forms of product labeling regulation in the United States were limited in impact, by modern standards, but set the foundation for future labeling regulation. The Sherley Amendment of 1912 prohibited “false and fraudulent” labeling, becoming the first federal law to regulate labeling based on manufacturer intent instead of contents [22].

In 1990, the Nutrition Labeling and Education Act gave the FDA the authority to require and regulate health labeling. This act also standardized and defined health benefit terms. The standardized format of the nutrition facts label was designed to limit the ability of a manufacturer to conceal certain facts while the standardized definitions of health-benefit terms prevented ambiguous claims [22].

While effective at conveying key information, the original ‘nutrition facts’ label format was found to be difficult to interpret for those with math skill deficiencies [23]. A revised design was developed which simplified the amount of math involved. Certain products were given new serving size suggestions with more realistic expectations, such as a 20 oz. bottle of soda now being regarded as one serving [24]. While some serving sizes were not ideal consumption recommendations, from a health perspective, it was seen as more important to clearly inform the consumer of how much sugar they were drinking than to try to persuade them to consume a soda across multiple servings.

In addition to the ‘nutrition facts’ labels, the federal government regulates consumer disclosures on numerous products and services. A discussion of these regulations can be found in [25].

Several of these forms of labeling are directly relevant to the labeling of online content. For entertainment, the MPAA and V-Chip ratings [26,27,28] (for movies and television, respectively) provide some benefits sought by those who seek to label online content. Both of these systems differ from most online content labeling due to their primary focus on age appropriateness, instead of content-targeted information and restrictions. However, in some cases, a description of the reason for the rating is provided which may provide some content insight.

Video games, including online games, utilize a conceptually similar system developed by the Entertainment Software Rating Board (ESRB). Each ESRB rating is comprised of a letter, a text description field and a supplemental interactive activity (e.g., in-game purchases) description field [29]. The ratings are assigned by reviewers (for boxed games) or from a survey that results in automatic rating assignment (for downloadable games) [30].

Across all of these areas, the U.S. federal government has established a role in ensuring that consumers receive accurate information and warnings. In content areas, warnings have been more limited and focused on protecting children and ensuring information availability and accuracy for commercial transactions. The more limited approach to content regulation is likely due to the influence of the First Amendment to the United States Constitution that states that “congress shall make no law … abridging the freedom of speech, or of the press” [31].

A number of labeling regulation issues are key topics of current interest. These include the labeling regulation of genetically modified organisms and nanotechnology products [32], the regulation and labeling of games which include microtransactions and “loot boxes” [33], and regulations that seek to label products based upon their environmental impact [34]. Different paradigms for labeling adoption have been proposed. Some suggest minimalist labels that present only the most crucial facts. Others suggest that, rather than simply addressing false advertising or immediate health risks, labeling can be adopted for cases where the public desires or can benefit from assistance in making informed choices about their purchasing or consumption habits. Labels that consider broader product impacts may allow consumers to avoid longer term, less obvious, or even incorrectly perceived risks associated with a product. While some of these labeling schemes apply to online advertisements and product sales, none specifically regulate news-style content.

### 2.2. Identification and Classification of Fake News

The 1936 International Convention of the Use of Broadcasting in the Cause of Peace codified that the use of misinformation to impact a foreign state violates the principles of non-intervention. It stated that every state has the right to conduct its internal affairs without outside interference and defined fake news as being “deliberately false and intended to produce dissent or encourage insurgents.” The convention states that “the High Contracting Parties mutually undertake to prohibit and, if occasion arises, to stop without delay within their respective territories any transmission likely to harm good international understanding by statements the incorrectness of which is or ought to be known to the persons responsible for the broadcast” [35]. Of course, the League of Nations did not perhaps foresee the speed at which modern mass media can report upon the daily news and just as quickly make mistakes. Furthermore, while fake news may come from a foreign state, the sources need not be international. Misinformation created by and for a single population can be just as dangerous.

In modern usage, the term “fake news” has been used to refer to satirical works such as “The Daily Show” and “The Onion” [36], images designed to spread misinformation in the form of “internet memes” [37], invented news organizations created for the purpose of making fake news stories appear to come from legitimate sources [38], and as a pejorative term for “news I do not like” [39].

Attempts have been made in recent years to codify a common definition of “fake news”, such as by the European Commission [40] and by UNESCO [41]. These definitions share the idea that fake news includes media serving as a form of disinformation (false information created to cause harm), misinformation (false information created without the intent to cause harm), and mal-information (uses selective true information organized in a manner to cause harm).

The labeling of intentionally deceptive online content necessarily begins with its identification. Lazer, et al. [4] define ‘fake news’ as “fabricated information that mimics news media content in form but not in organizational process or intent.” Gelfert [42], though, argues that the term fake news ought to be reserved only for deliberately misleading or manipulative content.

Fake news presents a problem, if not identified by a consumer and treated as real. Deceptive content creators, thus, have an incentive to make fake news identification as difficult as possible to fool consumers and identification software alike. The problem of fake news, in this regard, is conceptually similar to the phenomenon of ‘aggressive mimicry’ in nature, where a predatory or parasitic organism mimics a benign creature [43]. This is an apt parallel as, in most cases, fake news is designed to be consumed along with or in place of real news.

Tornero et al. [44] describe the fake news phenomenon as a symptom of a larger weakness in the safeguards of news media in general, noting that “the almost complete lack of filters and information verification systems is becoming apparent, and the classic criterion and procedures for news information verification have either disappeared or become so weak or powerless that they work insufficiently.” Deceptive content’s ability to spread over social media is so virulent that it has been compared to the spread of a virus [45].

The potential harm that fake news presents to the public is pronounced. A fake news story led to an armed standoff at a pizza parlor [6]. Deceptive content has been shown to have undermined efforts to educate the public about COVID-19 and vaccinations [46]. Numerous other examples exist.

To attempt to combat the impact of deceptive content, a number of educational initiatives related to “news literacy” have been conducted to attempt to help the public to identify fake news. Scheibenzuber et al. [47] proposed a framework for designing an undergraduate study in fake news literacy, while Bonnet and Rosenbaum [48] proposed a news literacy workshop format. Grace and Hone [49] studied the effectiveness of a game designed to teach fake news literacy, and found that it improved impact as the age of the players increased, up until age 70.

Several studies have noted that teenage players can have more difficulty differentiating between real news, entertainment, and deceptive content due to a tendency amongst this age group to reject classical news sources in favor of social media sources [50,51,52]. Literat et al. [53] suggested a “flipped” approach to the idea of the gamification of news literacy by involving students in the game design process. While the efficacy of several of these initiatives remains unclear, measures of this type can be crucial to the future of any deceptive content labeling system and may work in tandem with it and support its adoption similar to how nutrition facts labeling works is supported by health and nutrition science education in the United States [54]. None of the aforementioned studies, though, fully answer the question of how to label online content or how consumers will react to labeling.

### 2.3. Methods and Perceptions of Fake News Labeling

Research regarding warning labels and their impact on consumer decision making has been extensive, spanning over several decades. Topics of study have included consumer responses to toxic product warnings [55], cigarette warning labels [56], nutrition labeling for food products [57], and alcohol warning labels [58]. Modern work has considered the impact of political stance labeling and credibility labeling on the perceived trustworthiness of news articles, where it was found that fake news articles could be made to look more trustworthy when labeled as having a political stance the consumer agrees with [59]. Another recent study showed that fake news, when allowed to incubate over time without being flagged as fake, can create an “illusory truth effect” whereby the story becomes more believable over time [60].

Payloads of misinformation, selective information, emotional manipulation, or a combination of the aforementioned can be designed to drive counter-intuitive actions (see, e.g., [7,61]) by information consumers, who believe themselves to be acting in a well-informed and justified manner. This makes undoing the damage of fake news challenging, as the information consumers see their actions as being fully rational within their operating paradigm. Indeed, attempts to point out fake news may be ignored by those with low conscientiousness levels who may “desire to create chaos” [62], and others may simply not trust the warnings [63].

These issues are not without parallel, however. Victims of behavioral conditioning within cults are known to have trouble breaking away. Wright [64] observed that former cult members experience depression, loneliness, and dissociated states. Cult programming can make life after the cult more challenging whether the victim voluntarily leaves or is ‘deprogrammed’ with the aid of psychological professionals or other support groups.

While fake news exposure may not be as immersive or controlling as cult membership, individuals may, nevertheless, need assistance in their decision-making to avoid its effects. To attempt to combat the effects of fake news content, several studies have proposed standardized label designs for online media [25,65,66,67]. In prior work [68], two university populations in different regions of the United States were surveyed about different labeling styles and label content. Differences were identified in respondents’ perceptions based upon their age and other characteristics. A particular focus was to better understand differences seen within the “digital native” age group and between this group and others [68]. Digital natives are those at an age where their generation has been exposed to internet and, to a lesser extent, social media their entire lives. They show a greater ease in identifying deceptive news content [69] but also tend to trust social media more than traditional news sources [52].

Due to their heightened reliance on social media and imminent coming of age and to better understand the future impact of fake news, this group is of particular focus. Notably, digital natives have been shown to interact with media differently than older populations [67,69,70]. Thus, they may be reluctant to trust labeling information coming from a traditional source, perhaps preferring instead to rely upon their own intuition.

This prior work, across multiple areas of focus, lays the foundation for the analysis presented herein. Even the most closely related studies, though, fail to consider the entire U.S. population, which is key to fully understanding labeling decisions.

### 2.4. User and Industry-Initiated Labeling and Fact-Checking

Some labeling mechanisms have been implemented for certain social media platforms. Twitter labels, for example, can warn a user that a “tweet” contains misinformation related to the spread of COVID-19 and the safety of the vaccine [13]. Additionally, Twitter has released a feature called “Birdwatch” through which Twitter users themselves label misinformation they discover and then rate one another’s labels for their quality [70]. Facebook has, similarly, turned to a crowdsourced factchecking solution, where users may flag a post as needing fact-checking; if enough users flag a post, the company has the post analyzed by a third party for possible labeling [71].

In considering industry-initiated labeling, it is important to note that social media companies and news organizations are not neutral parties. Decisions on which features to include in a media labeling solution may not be solely based upon how well it works at mitigating misinformation. A common trend between Facebook and Twitter, for example, appears to be moving more of the load of mitigating misinformation from the social-media sites themselves to consumers. Neither Twitter’s Birdwatch nor Facebook’s latest crowdsourcing program could exist without volunteers. It remains a question as to what extent this solution will shield the companies from legal disputes over the quality of curation that occurs on their platforms. It also remains a question whether these solutions will be effective in mitigating the spread of fake news over these platforms. If much of the work is to be done by the users themselves, a cross-platform open-source tool could allow for more control and transparency over the design of the system.

While they remain shouldered with the burden of identifying fake news themselves, users may make use of fact-checking third party platforms. Multiple online fact-checking resources exist, including Factcheck.org (accessed on 23 October 2021), Factmata.com (accessed on 23 October 2021), PolitiFact.com (accessed on 23 October 2021) and Snopes.com (accessed on 23 October 2021). Additionally, Wikipedia volunteers maintain a database (https://en.wikipedia.org/wiki/Wikipedia:Reliable_sources/Perennial_sources, accessed on 23 October 2021) of news sources rated by their reliability, with some earning a “generally unreliable” or even “blacklisted” rating.

Industry participation will be key to implementing any labeling system. However, any such system can only be successful if information consumers trust and use it. The study presented herein can, thus, help inform industry and other efforts through providing greater knowledge regarding consumer perceptions and attitudes towards labeling.

## 3. Study Design

This section describes the survey that was used to collect the data that is presented and analyzed herein. First, the survey and its administration are discussed. Then, respondent characteristics are reviewed.

### 3.1. Survey and Administration

The results that are presented herein were collected using a quota-based stratified sampling technique implemented by Qualtrics International Inc. Respondents were recruited based on population-proportionate age, gender, household income, and political affiliation targets. Approximately 550 responses were collected that had answers to the questions analyzed in this paper. Of these, 500 were part of a nationally representative sample based on Qualtrics’ standard quota-based stratified sampling technique.

As respondents were given an incentive based on survey completeness, the vast majority of responses had answers to all of the questions analyzed herein. Respondents were recruited from across the United States. They survey was administered using Qualtrics during an approximately two-week period in October 2021. The characteristics of the respondents are presented in the subsequent section.

### 3.2. Respondent Characteristics

This section presents the demographic characteristics of the respondents who completed the survey discussed in this paper. Section 4 presents the results of a nationally representative sample, which utilized 500 of these responses that were complete and met the demographic quota targets discussed in that section. The remainder of the paper utilizes all of the responses that included answers to the relevant demographic question and the question being analyzed.

Respondents were asked to select their age group from 10 categories, ranging from 18–24 to 65 and older. The distribution of respondents’ ages is presented in Table 1.

Respondents were also asked to indicate their highest educational level, ranging from some high school being completed without graduating to attaining a doctoral degree. The distribution of respondents’ educational attainment levels is presented in Table 2.

Respondents also indicated their household income level by selecting from six categories ranging from below $25,000 to $125,000 or more. Respondents’ household income distribution is presented in Table 3.

Respondents were asked what political party they most closely identify with. They were able to indicate a preference for the Democratic Party, Republican Party or “independent/other party”. The respondents’ political affiliation distribution is presented in Table 4.

Finally, respondents were asked to identify their gender. The options presented included male, female, and non-binary (with the option to fill in a specific non-binary gender, if desired). Respondents’ gender distribution is presented in Table 5.

Due to the limited number of respondents indicating a non-binary gender, insufficient data was available to further analyze in terms of this demographic group. This is left as a key area of potential future work.

## 4. Overall Population Representative Results

Using the standard methodology [72] utilized by Qualtrics Panels (Extensive documentation exists regarding the process and methodologies used by Qualtrics International Inc. to conduct surveys. The justification for key foundational principles can be found in [73]), a nationally representative sample was conducted for the demographics of age, gender, household income, and political affiliation. Age, gender, and household income proportion levels were provided by Qualtrics and based off of the 2019 U.S. Census Bureau American Community Survey data [74]. This data did not include a proportion for non-binary gender respondents. Due to this, all responses received from non-binary gender respondents, meeting response completeness criteria, are included. Only three complete non-binary gender responses were received. Additionally, political affiliation proportion levels were sourced from Gallup poll data [75]. The characteristics of the nationally responsive sample are presented in Table 6 and statistical significance calculations for the data presented in this section, in the form of confidence intervals, are included in Appendix A.

This section presents the results for the nationally responsive sample. Subsequent sections conduct analysis based on age, gender, education level, household income, and political affiliation.

### 4.1. Data and Discussion

**Figure 1:**
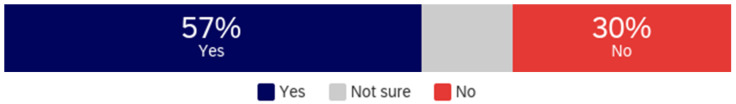
Have you ever seen a social media post with a warning label?

The data collected shows that most Americans have seen a social media post with a warning label and have familiarity with this topic. As shown in Figure 1, over half (57%) of respondents have seen a social media post with a warning label and only 30% have not. Approximately one-seventh (13%) of respondents were unsure whether they had seen a social media post with a warning label. Of those not indicating uncertainty, approximately two-thirds (66%) of respondents have seen a social media post with a warning label.

**Figure 2:**
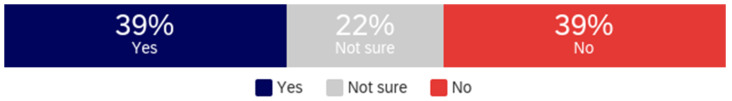
If you have seen a warning label on a social media post, did the warning label make you less likely to trust the contents of the post?

While more than half of respondents have seen a post with a warning label, the respondents are divided as to whether it impacted their perception of the post. Of those not indicating uncertainty, half said that the warning label had an impact on them and half said that it did not, as shown in Figure 2.

**Figure 3:**
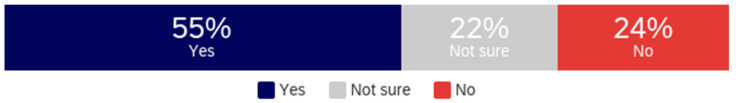
Do you think that warning labels make other people less likely to trust the contents of a post?

While respondents were equally split on the impact of warning labels on them, approximately 70% of respondents, who did not indicate uncertainty, indicated that they would make other people less likely to trust posts’ content. This is depicted in Figure 3. The difference between perceived efficacy on individuals’ own behavior and the behavior of others may indicate an overestimation of the impact on others.

**Figure 4:**
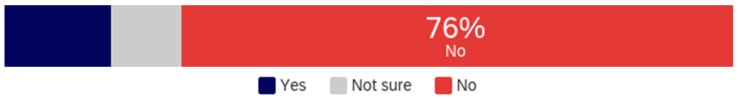
Has a warning label ever been attached to a post that you have made?

Respondents were then asked whether they had ever had a warning label attached to a post that they had made. As shown in Figure 4, only 15% of respondents indicated that a post they had made has had a warning label attached (10% were unsure).

**Figure 5:**
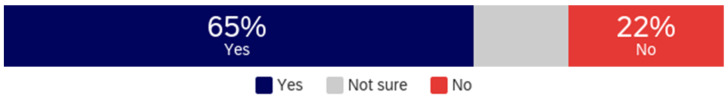
Should social media and news websites place warning labels on potentially misleading or false information?

Despite less than half of respondents indicating the efficacy of warning labels for their own use, 75% of respondents (who did not indicate uncertainty) thought that they should be applied to “potentially misleading or false information”. Less uncertainty existed regarding this as well, as only 13% of respondents indicated uncertainty regarding whether to label or not, as opposed to 22% being uncertain regarding labels efficacy at influencing their trust or the trust of others. This data is presented in Figure 5.

**Figure 6:**
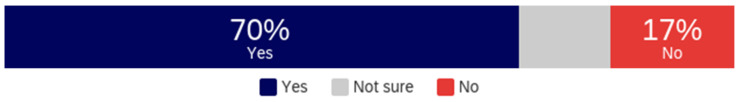
Should social media and news websites place warning labels on information that is potentially dangerous to your health?

Finally, as shown in Figure 6, agreement amongst respondents was even more pronounced with regards to placing warning labels on posts that are “potentially dangerous to your health”. Of the respondents who did not indicate uncertainty in this area, 80% indicated support for labeling. The level of uncertainty (13%) was similar to whether to place warning labels on misleading information.

### 4.2. Nationally Representative Sample Analysis

Among the survey respondents, 65% support the use of warning labels for informing readers about misleading or false information and 70% support warnings regarding health hazards. By comparison, presidential opinion polls frequently show approval rates in the 40% to 60% range [76,77]. Similarly, the Gallop poll approval rating for the US Congress has not exceeded 60% since 1974, except for the six months following the September 11th attacks [78]. With significant disagreement over what should be done and prioritization, areas of agreement to the level shown for warning labels are notable. The level of support indicated by respondents for media labeling is similar to the high level of agreement shown for criminal justice reform, which has been surveyed to be supported by between approximately 75% [79,80] and 95% [81] of Americans.

Notably, the level of agreement in support of the use of warning labels, particularly for medical-related warnings, far exceeds the size of any one group. The level of agreement between different demographic groups, with regards to these key questions, is assessed in the subsequent sections.

## 5. Analysis by Age

Age has the potential to be a key demographic characteristic with regards to interaction with social media. Younger respondents have potentially been exposed to social media during their childhood and adolescence. Slightly older age groups, while not having social media as a child, have had access to social media for all of their adult lives. Older respondents, though, would have been introduced to social media later in life and, consequently, may have different perceptions due to its later-stage introduction. This section analyzes survey responses by age group. Statistical significance calculations (confidence intervals) for this data and analysis are included in Appendix A.

First, respondents were asked whether they had ever seen a social media post with a warning label. Responses range from approximately 75% of 25- to 29-year-olds saying that they have seen a warning labeled post to just over 30% of those 65 and over saying that they have seen one. There is a notable trend where younger respondents are more likely to indicate that they have seen a warning label tagged post, with approximately 80% to 85% of those in the 18–29 age groups indicating that they have seen one (excluding those indicating that they are unsure), 60% to 70% in the 30 to 64 age groups and approximately 35% of those 65 and older indicating that they had seen one.

In addition to the overarching trend, there appear to be five cohorts within this data. The first is the most familiar with and likely to have seen a warning label: respondents between 18 and 29. There is a second cohort between 30 and 39, which has a lower level of warning label exposure than the groups on either side of it. A third cohort group is located at 40–49 with greater exposure to warning labels than the groups on either side of it. A fourth cohort group can be found between 50 and 65, which has notably lower warning label exposure than the 40–49 cohort group but also notably higher warning label exposure, as compared to the 65 and older cohort group.

**Figure 7:**
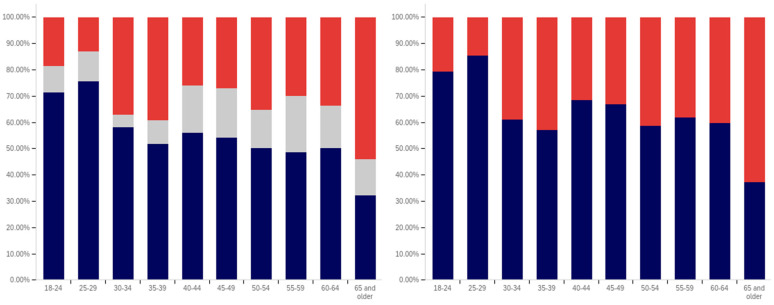
Have you ever seen a social media post with a warning label?—responses by age group, including uncertain responses (**left**) and excluding uncertain responses (**right**).

One potential explanation for the drop off in usage in the 30–39 age cohort is a transition into professional maturity. A study of social media use in 2021 [82] showed a significant drop in usage of Snapchat (−41%), TikTok (−26%), Instagram (−23%), Twitter (−15%), and Reddit (−14%) when transitioning from age 18–29 to age 30–49. At the same time, there was an increase in usage of Nextdoor (+12%), Facebook (+7%), LinkedIn (+6%), and WhatsApp (+6%). Combined with the results presented in Figure 7, this suggests that the 30–39 and 40–49 cohorts begin to use social media with less frequency while also transitioning to platforms focused upon professional, residential, and family networking.

Also interesting is the increasing level of uncertainty. In the 18 to 39 age groups, approximately 10% or less of respondents indicate uncertainty regarding whether they have seen a warning labeled post, with a peak minimum of less than 5% within the 30–34 age group. However, for respondents in the 40-year-old and older age groups, the uncertainty level is 20% or more.

Clearly, there is an age correlation between awareness and understanding of social media post warnings. Additionally, the data suggests that warnings may be more likely to be present on social media frequented by younger individuals.

**Figure 8:**
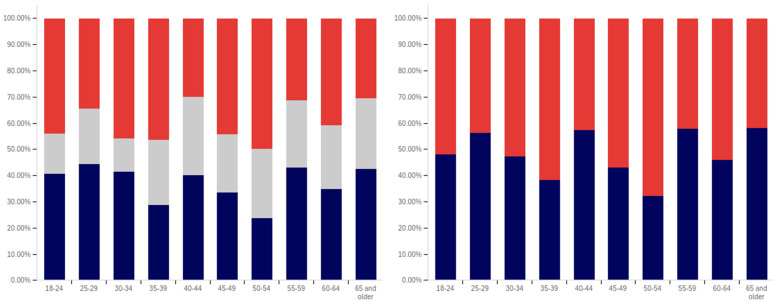
If you have seen a warning label on a social media post, did the warning label make you less likely to trust the contents of the post?—responses by age group, including uncertain responses (**left**) and excluding uncertain responses (**right**).

Respondents were next asked whether seeing a warning label made them less likely to trust a post. This data is presented in Figure 8. There is not a clear age-related trend that spans all of the data, instead waves of decreased trust appear to be present in different age groups. This may be due to differences in the socialization of the population over time (with different ages having different perspectives on trust). Notably, the three youngest groups indicate amongst the highest levels of warning impact, with all three indicating that around 50% of respondents (excluding those that are uncertain) would have reduced trust in posts with warning labels. Notably, the 40–44, 55–59, and 65 and older age groups have higher percentages of individuals indicating reduced trust, as compared to the 18 to 34-year-olds; however, their adjacent age groups indicate much lower levels of impact.

It is also notable that, while the overall levels of uncertainty reported are higher than for the previous question regarding seeing a warning label, the trend of the uncertainty being higher for older age groups is again present. The 30–34 age group has the lowest uncertainty, at approximately 10%, while over a fifth of the 65 and older age group indicates uncertainty.

**Figure 9:**
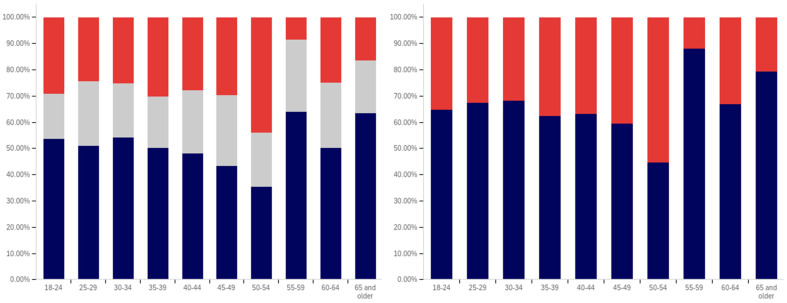
Do you think that warning labels make other people less likely to trust the contents of a post?—responses by age group, including uncertain responses (**left**) and excluding uncertain responses (**right**).

Following the question about their own response to warning labels, respondents were asked whether they felt that warning labels were likely to reduce others’ trust in social media posts. This data is presented in Figure 9.

**Figure 10:**
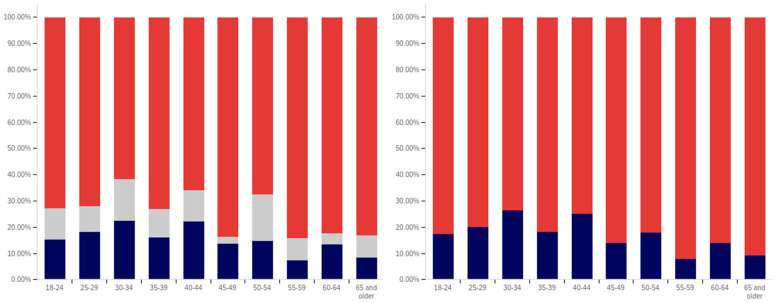
Has a warning label ever been attached to a post that you have made?—responses by age group, including uncertain responses (**left**) and excluding uncertain responses (**right**).

Comparing the data presented in Figure 9 and Figure 10, more respondents, across all age groups, indicated that they believed that warning labels would cause others to have less trust in posts than indicated that they themselves would have reduced trust in posts due to them. In many cases, the difference was around 20% of respondents. A less pronounced trend of greater uncertainty being indicated by those with advanced age is, again, present.

What is perhaps most interesting from this data is that there is a notable decline in the perceived efficacy of warning labels on others, with consistent decreases starting from between the 30–34 and 35–39 age groups. However, there is an abrupt surge in perceived efficacy amongst others—to the highest level of any age group—in the 55–59-year-olds. Notably, this is an age associated with the ability to begin retirement (without certain penalties [83]) and certain senior discounts ([84]), though some discounts [85] and retirement eligibility [83] appears to begin at 50. Given the correlation with senior citizen status and retirement preparation, this may indicate the efficacy of senior-targeted awareness campaigns. While this trend is more pronounced with the efficacy in others question, a similar—albeit less pronounced—trend is also shown in response to the self-efficacy question (as shown in Figure 9).

Next, respondents were asked about whether posts they had made had ever been warning labeled. Warning labeling was reported most pronouncedly amongst the 18-to-44-year-olds, peaking in the 30–34-year-old age group (with a second peak in the 40–44-year old age group). Notably, the older age groups from (45 and older) report lower levels of their posts being warning labeled. Additionally, the trend of greater uncertainty with age is not present in these responses. The lowest uncertainty levels are reported in the 45–49-year-olds and 60–64-year-olds age groups. All age groups report less than 25% of respondents knowing their posts have been warning labeled, so most respondents have not experienced their personal posts being warning labeled.

**Figure 11:**
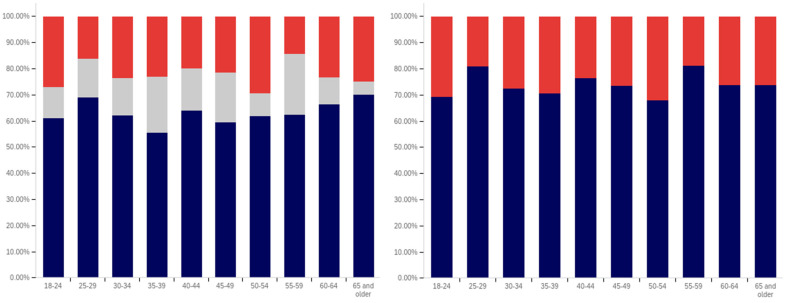
Should social media and news websites place warning labels on potentially misleading or false information?—responses by age group, including uncertain responses (**left**) and excluding uncertain responses (**right**).

**Figure 12:**
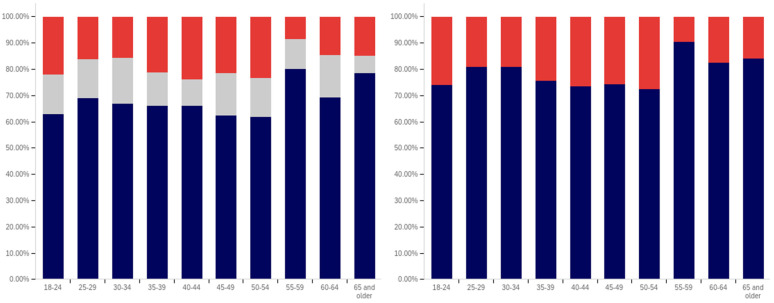
Should social media and news websites place warning labels on information that is potentially dangerous to your health?—responses by age group, including uncertain responses (**left**) and excluding uncertain responses (**right**).

Next, respondents were asked two questions regarding whether they felt that certain types of posts should be labeled. This data is presented in Figure 11 and Figure 12. First, respondents were asked whether they felt that that social media operators should place labels on potentially misleading or false posts. Over 60% of respondents in all age groups (ignoring those indicating uncertainty) indicated support for warning labeling these posts. For most age groups, over 70% indicated support. Unlike several of the earlier questions, where uncertainty increased by age, uncertainty showed a general trend of decreasing with age, in regard to this question. The three lowest uncertainty levels were reported in three of the four oldest age groups (albeit with the highest uncertainty level being reported in the last, the 55–59-year-olds group).

The response to the subsequent question regarding placing warning labels on potentially dangerous-to-health posts was even more pronouncedly supportive, in most age groups. Over 70% of respondents, in all groups, supported labeling dangerous-to-health posts and in all but one age group (40–44), the level of support was approximately the same or higher than for labeling misleading or false (non-health-related) posts. Two groups (30–34 and 55–59) showed an 9% (of respondents) increase, one group (60–64) showed an 8% increase and one group (65 and older) showed a 10% increase. No clear correlation regarding uncertainty and age was present in this data.

From the foregoing, it is clear that there are notable correlations between deceptive media awareness, perceptions of labeling efficacy and labeling support, and age levels. Also notable are the age-correlated differences in uncertainty levels reported.

## 6. Analysis by Education Level

Survey responses are now analyzed by educational attainment level. Respondents ranged in educational completion from those having attended but not completed high school to individuals with doctoral degrees. As were present with age, a number of educational attainment-correlating trends are shown. Confidence interval calculations for this data and analysis are included in Appendix A.

Notably, there will be some correlation between age and educational attainment, as younger groups will typically not have had time to complete advanced degrees. For example, the 18–24 age range is unlikely to have completed education at a higher level than ‘Bachelor’s degree’ due to the traditional ages of college students in the United States falling within this range. Some 18- and 19-year-olds may also still be attending high school. This overlap is not complete, though, as the group will contain both those who have dropped out and those who are still pursuing their education. Associate’s degree, bachelor’s degree, and master’s degree groups may, similarly, include some who have attained this credential and are pursuing further education, as well.

**Figure 13:**
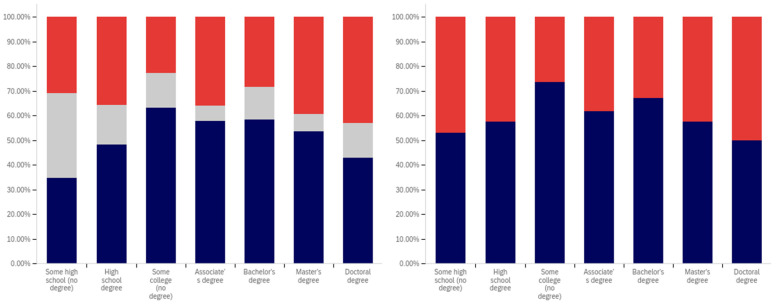
Have you ever seen a social media post with a warning label?—responses by education level, including uncertain responses (**left**) and excluding uncertain responses (**right**).

The first question, whether respondents have seen a warning labeled social media post, shows two interesting patterns (shown in Figure 13). Up to the ‘some college’ educational level, the percentage of respondents reporting seeing warning labeled posts increases. From the bachelor’s degree level upwards, respondents report seeing warning labeled posts with less frequency. Also notably, the some high school and high school degree and some college groups report three of the four highest levels of uncertainty (the third highest level is reported by doctoral degree holders).

**Figure 14:**
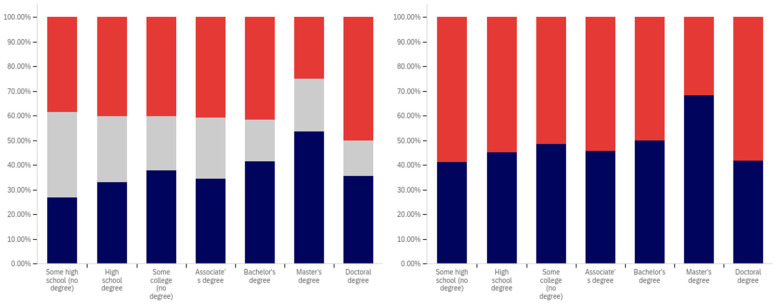
If you have seen a warning label on a social media post, did the warning label make you less likely to trust the contents of the post?—responses by education level, including uncertain responses (**left**) and excluding uncertain responses (**right**).

Limited correlation between educational level and reduced post trust for warning labeled posts was shown between the “some high school”, high school degree, “some college”, and bachelor’s degree groups, as shown in Figure 14. A large jump exists between the bachelor’s degree holder and master’s degree holder level, with approximately 20% more of master’s degree holders indicating reduced trust when warning labels are present. Interestingly, this pattern does not continue with doctoral degree holders who report the second least trust diminishment due to warning labels of all groups (the least trust diminishment is reported by the “some high school” group).

Also notable is the trend in the reported uncertainty levels. Reported uncertainty, generally, goes down with increased educational attainment. The highest uncertainty level is reported by the ‘some high school’ group (35%) and the lowest (14%) is reported by doctoral degree holders.

**Figure 15:**
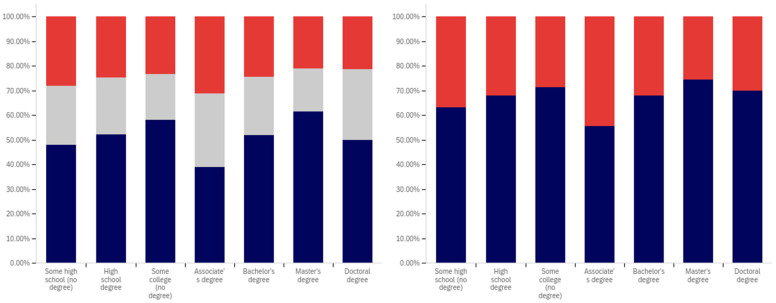
Do you think that warning labels make other people less likely to trust the contents of a post?—responses by education level, including uncertain responses (**left**) and excluding uncertain responses (**right**).

Another weak trend is present between respondents’ education level and the perceived efficacy of warning labels for others. This is shown in Figure 15. Slight increases are seen between the “some high school”, high school degree, and “some college” categories. The associate’s degree has the lowest level of perceived warning label efficacy in others. The bachelor’s level is close to, but marginally lower than the “some college” level. Another (albeit much less pronounced) peak is seen at the master’s level, such as with the previous question, with the doctoral degree holder respondents reporting lower perceived efficacy in others (mirroring their lower reported self-efficacy). No clear trend is noticeable with the uncertainty level data for this question.

**Figure 16:**
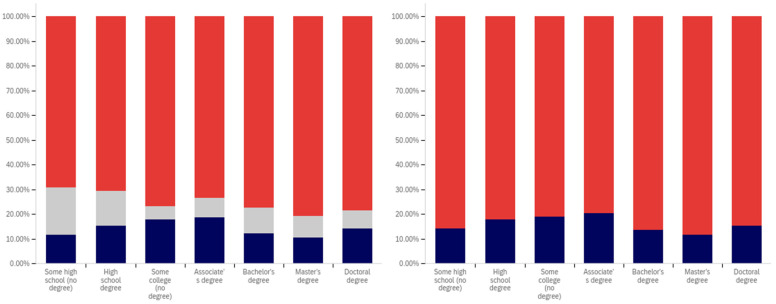
Has a warning label ever been attached to a post that you have made?—responses by education level, including uncertain responses (**left**) and excluding uncertain responses (**right**).

Respondents were next asked whether their posts had ever been warning labeled. This data is shown in Figure 16. Most respondents said that they had not ever had a post labeled. A pattern mirroring the previous two questions, again less pronounced than the trend shown in Figure 14, is present in this data. The percentage of individuals reporting labeling increases from group-to-group between the “some high school”, high school degree and “some college” groups. Unlike with previous questions, the associate’s degree holders report increased labeling as compared to the previous three categories. Bachelor’s degree holders report less warning labeling than both the “some college” and associate’s degree groups. A very small decline is also present between bachelor’s and master’s degree holders (which is, perhaps, most notable in that it is a decline instead of a peak). Doctoral degree holders report greater post warning labeling than both bachelor’s and master’s degree holders.

The trend regarding uncertainty is also less notable in this data. The two highest uncertainty levels are reported by those with the least educational attainment (‘some high school’ and high school degree). However, the trend does not continue from there, with the lowest level of uncertainty being reported by the ‘some college’ group.

**Figure 17:**
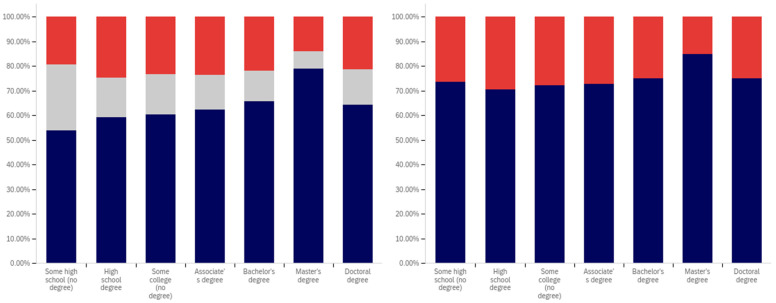
Should social media and news websites place warning labels on potentially misleading or false information?—responses by education level, including uncertain responses (**left**) and excluding uncertain responses (**right**).

Respondents were next asked about their support for labeling misleading and false pots. This data is presented in Figure 17. Over 70% of respondents (excluding uncertain answers) supported warning labeling for this content.

A very marginal group-over-group increase is seen between the high school degree, “some college”, associate’s degree, and bachelor’s degree groups. Master’s degree holders are a notable spike in supporting labeling, with doctoral degree holders returning to approximately the same level as the bachelor’s degree holders.

Notably, this data shows fairly consistent declines in uncertainty level with increasing educational attainment, up to and including at the master’s level. Doctoral degree holders indicate greater uncertainty (similar in magnitude to the associate’s degree level). Just as the level of support spiked amongst master’s degree holders, the level of uncertainty is minimal for this group as well. Master’s degree holders report less than half the level of uncertainty seen in any other group on this question of support.

**Figure 18:**
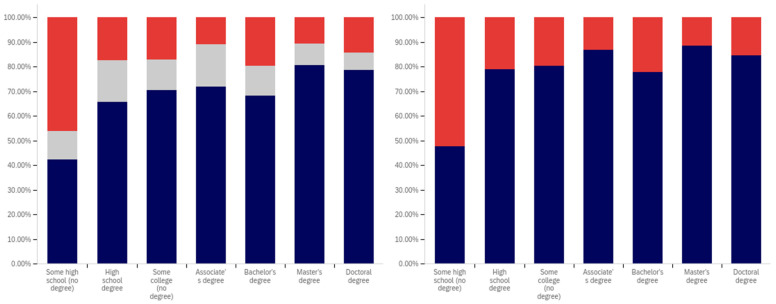
Should social media and news websites place warning labels on information that is potentially dangerous to your health?—responses by education level, including uncertain responses (**left**) and excluding uncertain responses (**right**).

Analysis, in terms of education level, of the last question presents one of the most interesting findings of this study. This data is shown in Figure 18.

In many ways, the education level-correlated support for warning labeling health-dangerous posts mirrors the support for misleading and false posts and the age-correlated and nationally representative analysis. Looking at the data that discounts uncertain responses, a marginal increase in support for warning labeling is seen between high school graduates and those with some college. A small peak exists at associate’s degree holders, with a larger (6%) increase between “some college” and associate’s degree holders than between previous levels. Support drops 9% between associate’s degree and bachelor’s degree holders, before increasing 10% between bachelor’s and master’s degree holders. Again, doctoral degree holders report a lower level of support than master’s degree holders (though notably higher than bachelor’s degree holders).

Unusually, compared to the data presented in the five previous figures and the overall study trends, the “some high school” group reports significantly less support for health-dangerous post labeling, with only approximately 40% of respondents supporting health-danger labeling (and less than 50% of not unsure respondents indicating support). While, generally, there is greater support for health-dangerous post warning labeling, the opposite is true for the “some high school” group.

Some of this significant difference may be attributable to the current COVID-19 pandemic and its impact on high school age students. Many in this educational level have been forced to attend school virtually for at least several months, while the disease has not typically affected their age demographic medically as severely as older ones. A variety of negative impacts have been associated with the age group’s limited social interactions during COVID-19 [86,87]. However, because the pattern of impact on the youth was not present in this question’s 18–24-year-old group data, this may not be a full (or correct) explanation. It could be that the impact of virtual school participation is particularly pronounced amongst those still in high school (thus not impacting the 20–24-year-olds, and thus the age category, as much). However, this could also be indicative of distrust of medical warnings amongst those with the lowest educational levels amidst the COVID-19 pandemic.

## 7. Analysis by Income Level

The focus of analysis now turns to income level-based correlations. There are certainly examples of individuals of all ages and educational levels earning amongst the highest incomes nationally (both Bill Gates and Mark Zuckerberg, for example, would still fall into the “some college” educational attainment category and both had significant earnings while still in their 20s). However, there clearly is a logical correlation between age and income level (as many people’s level of responsibility and pay increases with their experience) and educational and income levels (with higher paying jobs, in many cases, requiring at least a bachelor’s degree). Given this, some correlation with the results presented in the two previous sections is to be expected. Income level correlations are analyzed in this section and statistical significance calculations (confidence intervals) for this data and analysis are included in Appendix A.

**Figure 19:**
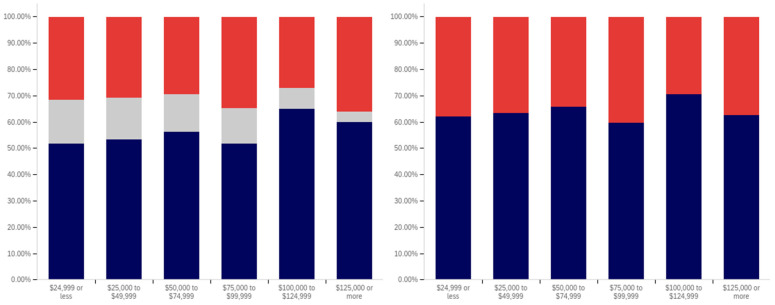
Have you ever seen a social media post with a warning label?—responses by income level, including uncertain responses (**left**) and excluding uncertain responses (**right**).

In analyzing the correlation between income level and having seen a social media warning label, Figure 19 shows that there is a slight increase between each successive level within the below $25,000 income level, the $25,000 to $49,999 income level, and the $50,000 to $74,999 income level data. There is also a slightly larger increase between the $50,000 to $74,999 and $100,000 to $124,999 levels (though the intervening $75,000 to $99,999 level reports lower levels of seeing social media post warning labels).

The level of uncertainty shows a trend of reduction correlating with increased income level, with the lowest income levels having between 15 and 20% uncertainty and the highest income level having approximately 5%.

**Figure 20:**
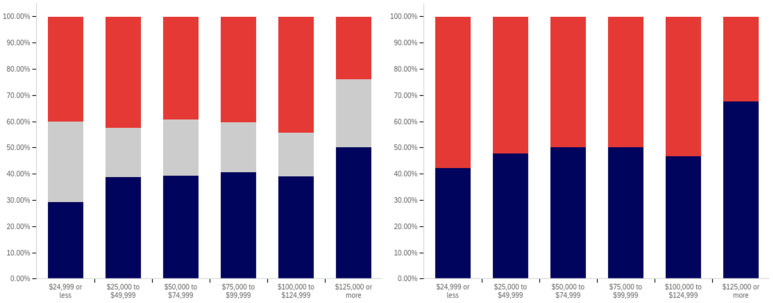
If you have seen a warning label on a social media post, did the warning label make you less likely to trust the contents of the post?—responses by income level, including uncertain responses (**left**) and excluding uncertain responses (**right**).

For warning label self-efficacy, as shown in Figure 20, a trend of limited increases in label effectiveness is shown between the less than $25,000 and $99,999 income levels. The $100,000 to $124,999 shows a slight decrease and then there is a significant (over 20%, when ignoring the uncertain) increase at the $125,000 or more level. Uncertainty for this question shows a general trend of reduction between the under $25,000 and $124,999 income levels. However, there is a significant jump in uncertainty (approaching the under $25,000 level) at the $125,000 and above income level. Thus, the two ends of the income spectrum have the two highest uncertainty levels.

**Figure 21:**
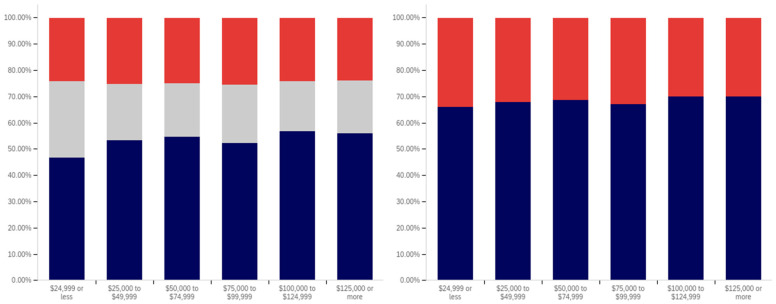
Do you think that warning labels make other people less likely to trust the contents of a post?—responses by income level, including uncertain responses (**left**) and excluding uncertain responses (**right**).

In comparing income level and perceived warning level efficacy for others (shown in Figure 21), very minimal changes are present with only slight increases that correlate with higher income levels (and a slight drop at the $75,000 to $99,999 level). There is a notable decrease in uncertainty between the under $25,000 level and the $25,000 to $49,999 level; however, only slight changes are present between higher income levels. Perceived efficacy for others of labeling, amongst those not indicating uncertainty, is, thus, fairly consistent at around 65% believing it to be effective.

**Figure 22:**
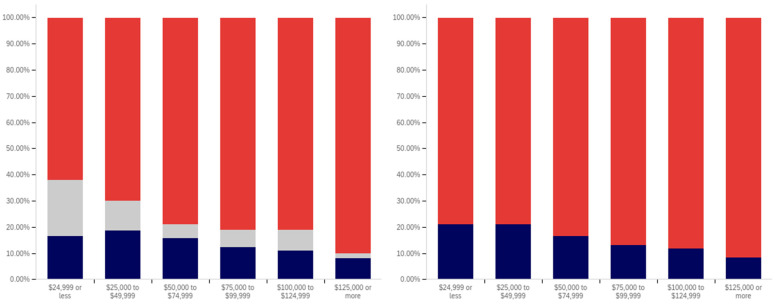
Has a warning label ever been attached to a post that you have made?—responses by income level, including uncertain responses (**left**) and excluding uncertain responses (**right**).

Unlike with the previous question, a notable negative correlation exists between higher income levels and lower rates of posts being warning labeled. As shown in Figure 22, the under $25,000 and $25,000 to $49,999 income levels have similar (and the highest) post warning labeling rates. Each successive higher income level, beyond these, has a decrease in warning labeling rate over the previous income level. Uncertainty levels also trend downwards with income level. While income level correlates with age and education (which both have similar trends), it is likely that individuals in more visible and responsible (and thus higher income) positions are also more careful with their social media posts. Given that, in addition to maturity and educational factors, there likely is an independent association between income and reduced warning labeling, as well.

**Figure 23:**
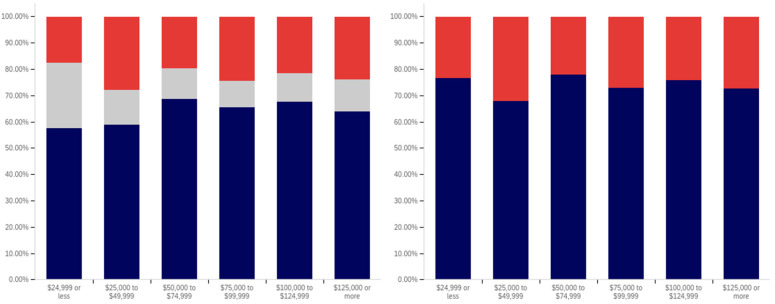
Should social media and news websites place warning labels on potentially misleading or false information?—responses by income level, including uncertain responses (**left**) and excluding uncertain responses (**right**).

There is no correlation between income level and support for labeling potentially misleading or false information that is notable in the data presented in Figure 23. Small differences, trending in both directions, are present between income levels; however, no general trend is present. Uncertainty is lower at the four higher income levels than at the lowest two; however, no trend of continued decrease exists within these four highest income levels. Generally, all income levels can be taken to be supportive of labeling, with approximately 70% or more of respondents in each (excluding those expressing uncertainty) indicating support for labeling.

**Figure 24:**
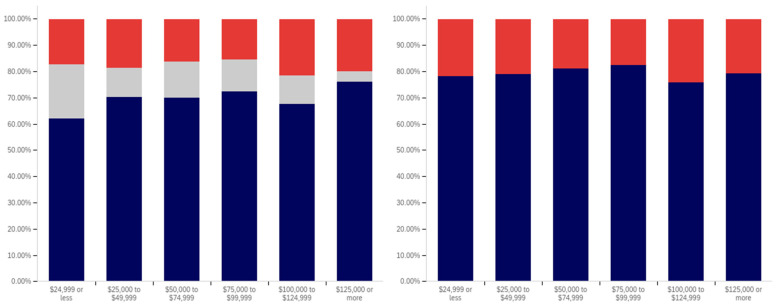
Should social media and news websites place warning labels on information that is potentially dangerous to your health?—responses by income level, including uncertain responses (**left**) and excluding uncertain responses (**right**).

In comparing the income level support for labeling potentially misleading and false information and potentially medically harmful information (shown in Figure 24), the general trend of greater support for labeling medically harmful information (as opposed to simply false or misleading information) is present across income levels as well. A marginal trend of increasing support between the under $25,000 and $99,999 income levels is present, but practically insignificant. The $100,000 to $124,999 income level has the lowest level of support, with the $125,000 or more returning to the under $25,000 to $49,999 level. The highest (and a notably higher) level of uncertainty is present at the under $25,000 level; however, no general trend of reduced uncertainty with increased income levels is noticeably present and general fluctuation of the exact values is shown.

## 8. Analysis by Political Party Affiliation

Analysis of respondents’ political party affiliation and their perceptions of deceptive content and warning labels could be expected to be one of the most controversial portions of this article. Those from both ends of the political spectrum have raised concerns about individuals on the other side. Political affiliation-based analysis is presented in this section and confidence intervals for this data and analysis are included in Appendix A.

**Figure 25:**
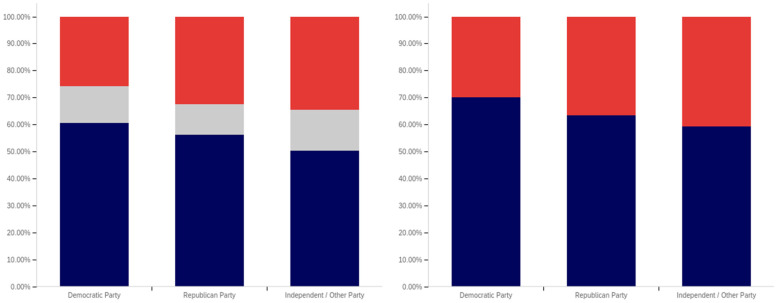
Have you ever seen a social media post with a warning label?—responses by political affiliation, including uncertain responses (**left**) and excluding uncertain responses (**right**).

This analysis begins, like in previous sections, with analyzing whether a correlation exists between individuals having seen a warning label and political affiliation. This data is presented in Figure 25.

The data shows that those reporting a Democratic Party affiliation also reported having seen warning labels the most (70%, excluding those indicating uncertainty), followed by Republicans (63%) and then independents (59%). Independents had the highest percentage of respondents indicating uncertainty (15%), followed by Democrats (14%) and then Republicans (11%).

**Figure 26:**
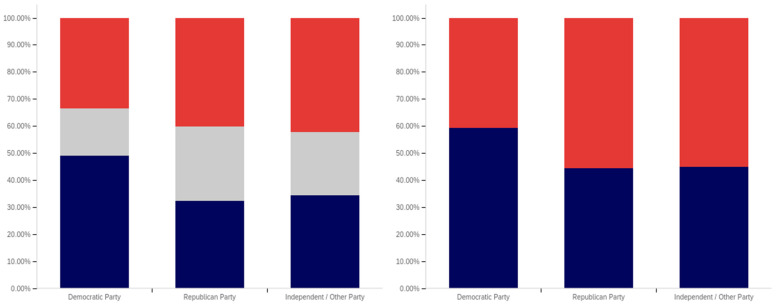
If you have seen a warning label on a social media post, did the warning label make you less likely to trust the contents of the post?—responses by political affiliation, including uncertain responses (**left**) and excluding uncertain responses (**right**).

In analyzing the self-efficacy of warning labeling (shown in Figure 26), it is notable that Democrats indicate the highest level of effectiveness, followed by independents and then (with a very marginal difference between them) Republicans. Excluding individuals expressing uncertainty, there is a 15% (of respondents) difference between Republicans and Democrats. Democrats indicate the lowest level of uncertainty (17%) about self-efficacy, while independents (23%) and Republicans (28%) indicate significantly more.

**Figure 27:**
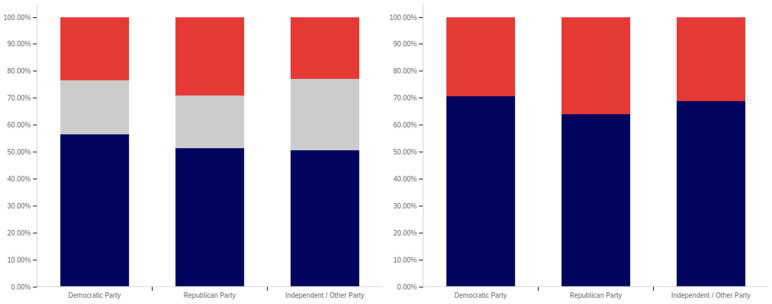
Do you think that warning labels make other people less likely to trust the contents of a post?—responses by political affiliation, including uncertain responses (**left**) and excluding uncertain responses (**right**).

There are also notable differences between Democrats and Republicans when analyzing the perceived efficacy of warning labels on others (shown in Figure 27). Excluding those indicating uncertainty, Democrats indicate the greatest perception of labels having efficacy for others. The perceived efficacy for others reported by independents falls between that of Republicans and Democrats, with Republicans indicating the lowest level and Democrats the highest. A 7% gap exists between Republicans and Democrats for belief in the efficacy of labels for others. Independents, notably, have a higher level of uncertainty regarding perceived warning label efficacy for others, as compared to both Republicans and Democrats, whose levels are not notably different.

**Figure 28:**
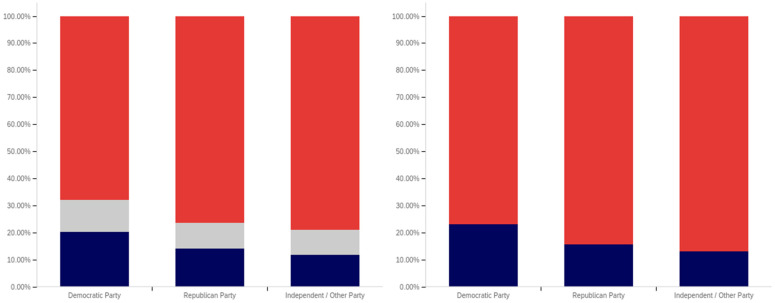
Has a warning label ever been attached to a post that you have made?—responses by political affiliation, including uncertain responses (**left**) and excluding uncertain responses (**right**).

In comparing political party affiliation and whether individuals’ posts have been warning labeled (as shown in Figure 28), 7% more (of respondents, excluding those indicating uncertainty) indicating a Democratic Party affiliation indicated having their post warning labeled than Republicans. Approximately 3% more of Republicans indicated having their posts warning labeled than independents. Democrats also indicated the greatest uncertainty (12%) as compared to Republicans and then independents (both slightly over 9%).

While those indicating a Democratic Party affiliation indicated the highest level of receiving warning labels on their posts, this does not necessarily correlate with posting greater levels of deceptive or harmful content. The increased support for the use of labels, discussed subsequently, may make Democrats more apt to report and label posts of concern, resulting in a higher level of labeling in areas frequented by Democrats. Differences in the perceived utility of warning labels between groups may also result in different levels of awareness of one’s own posts being labeled and thus knowledge of and willingness to self-report posts having been warning labeled.

**Figure 29:**
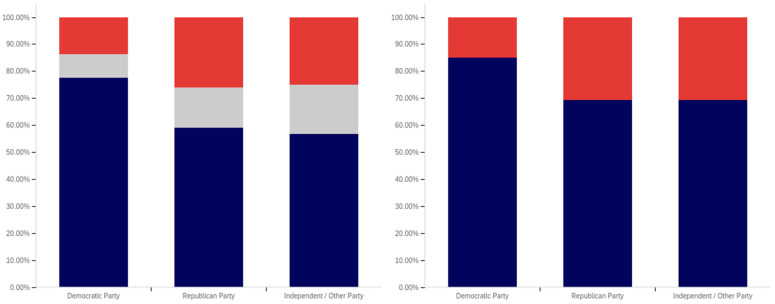
Should social media and news websites place warning labels on potentially misleading or false information?—responses by political affiliation, including uncertain responses (**left**) and excluding uncertain responses (**right**).

While approximately 70% or more of respondents from all three groups (Democrat, Republican, and independent/other party, excluding those indicating uncertainty) indicated support for labeling potentially misleading or false content, a notable difference between the levels of support by affiliation exists. This data is presented in Figure 29. For both independents and Republicans, 69% of respondents (ignoring those indicating uncertainty) indicated support for labeling. Democrats, however, had far more pronounced support with over 85% of respondents indicating support for warning labels for potentially misleading and false content—a 16% (of respondents, excluding those indicating uncertainty) greater level than Republicans and independents. Democrats had the lowest level of uncertainty about supporting labeling, as well, with only 9% of respondents indicating uncertainty, as compared to 15% of Republicans and 18% of those indicating an independent or other party affiliation.

**Figure 30:**
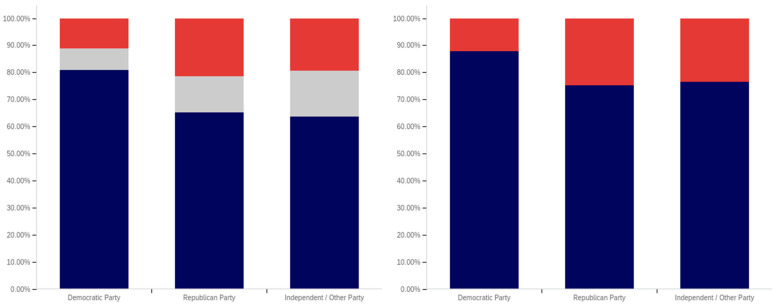
Should social media and news websites place warning labels on information that is potentially dangerous to your health?—responses by political affiliation, including uncertain responses (**left**) and excluding uncertain responses (**right**).

All three groups (Democrats, Republicans and those with independent/other party affiliations) indicated greater support for labeling medically harmful information, as compared to information that is just potentially misleading or false. This data is presented in Figure 30. Republican support increased 6% to 75% (of respondents, excluding those indicating uncertainty). Independent support increased 8% to 77%. Democrats’ support increased the least (3%), though still having the highest level of support for medically harmful content labeling at 88%. Like with the previous data, Democrats had the lowest level of uncertainty (8%). This was followed by Republicans (13%) and those with an independent/other party affiliation (17%).

## 9. Analysis by Gender

Analysis now turns to whether any gender correlation between perception of and support for warning labeling exists. This analysis is presented in this section and statistical significance calculations (confidence intervals) for this data and analysis are included in Appendix A. Note that, due to the limited number of non-binary responses received, only male and female responses are compared in this section.

**Figure 31:**
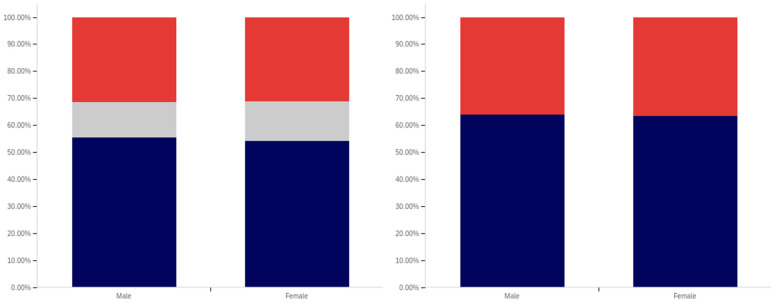
Have you ever seen a social media post with a warning label?—responses by gender, including uncertain responses (**left**) and excluding uncertain responses (**right**).

As shown in Figure 31, parity exists between males and females with regards to having seen a social media post with a warning label. The level of uncertainty between males and females is also very similar.

**Figure 32:**
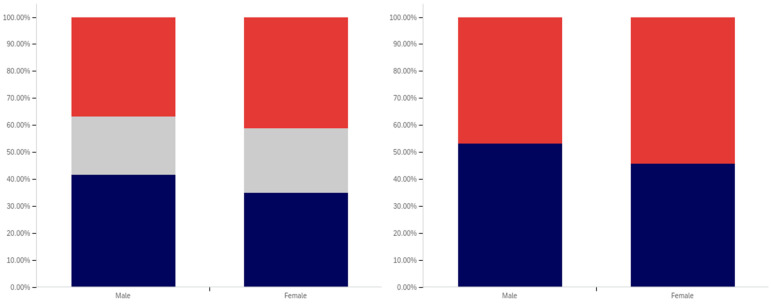
If you have seen a warning label on a social media post, did the warning label make you less likely to trust the contents of the post?—responses by gender, including uncertain responses (**left**) and excluding uncertain responses (**right**).

In comparing male and female respondents, as shown in Figure 32, males reported more warning label self-efficacy, with (excluding those indicating uncertainty) 53% of males indicating that they would be less likely to trust a labeled post versus 46% of females. Female respondents indicated uncertainty with slightly more frequency than male respondents with 24% indicating uncertainty versus 22% of males.

**Figure 33:**
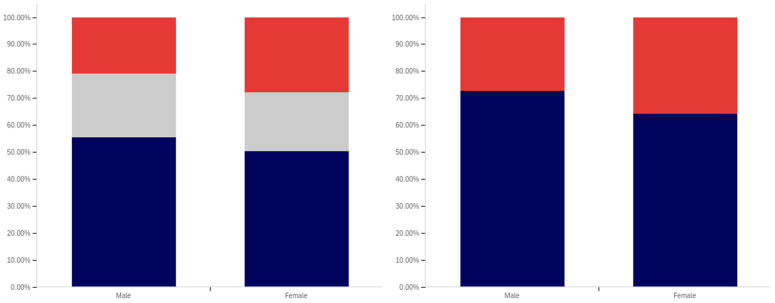
Do you think that warning labels make other people less likely to trust the contents of a post?—responses by gender, including uncertain responses (**left**) and excluding uncertain responses (**right**).

Male respondents, as shown in Figure 33, also indicated greater perceived efficacy of warning labels for others, with 73% of males indicating the belief that labels would be effective for others, versus 64% of females. Notably, since there was no indication of others’ gender, this would appear to suggest that males perceive warning labels as more effective for both genders, not just for other males. For this question, males indicated uncertainty with a greater frequency (24%) than females (22%).

**Figure 34:**
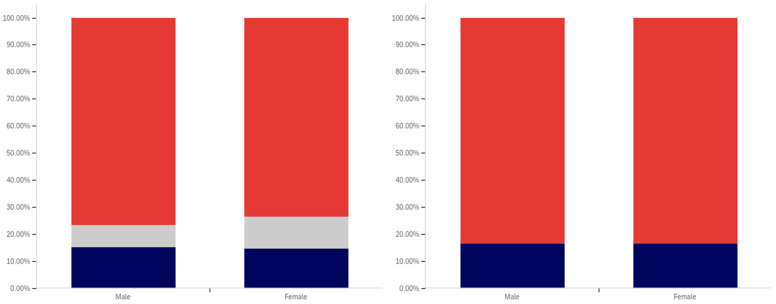
Has a warning label ever been attached to a post that you have made?—responses by gender, including uncertain responses (**left**) and excluding uncertain responses (**right**).

Males and females reported the same low level of application of warning labels to their posts. This data is shown in Figure 34. For both genders, 16% of respondents reported having had a warning label applied to one of their posts. Females indicated uncertainty with a slightly greater frequency (12%) for this question, as compared to males (8%).

**Figure 35:**
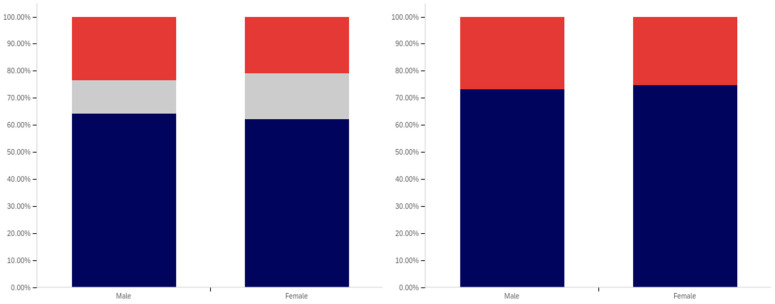
Should social media and news websites place warning labels on potentially misleading or false information?—responses by gender, including uncertain responses (**left**) and excluding uncertain responses (**right**).

Males and females indicated support for using warning labels for potentially misleading or false information with similar levels of frequency, as shown in Figure 35. Despite, as discussed above, indicating a greater perception of self-efficacy and efficacy for others, 73% of males indicated support for their use as opposed to 75% of female respondents. Females also indicated uncertainty with a slightly higher frequency (17%), versus males (12%).

**Figure 36:**
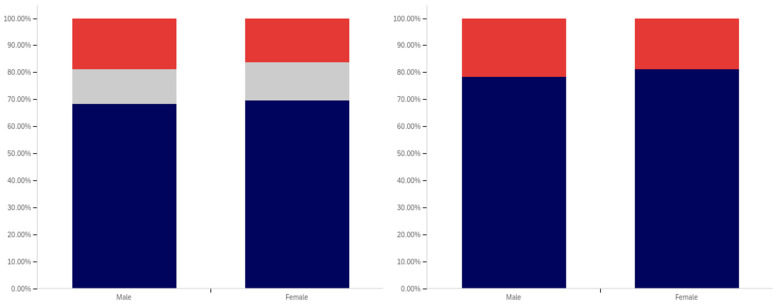
Should social media and news websites place warning labels on information that is potentially dangerous to your health?—responses by gender, including uncertain responses (**left**) and excluding uncertain responses (**right**).

Both male and female respondents, as shown in Figure 36, supported labeling medically harmful posts with a higher frequency than those that are just potentially misleading or false. Female respondents reported support with a 6% higher frequency of 81% (excluding those indicating uncertainty), while male respondents reported supporting labeling with a 5% higher frequency of 78%. Both genders indicated uncertainty with the same frequency (6%).

Interestingly, while both genders indicated strong support for labeling, males indicated notably greater perceived self-efficacy and efficacy of labels for others. Despite this, male respondents supported their use slightly less than female respondents.

## 10. Comparative Analysis

Previous sections of this article have analyzed the perspectives of a nationally representative sample of the population and conducted analysis in terms of key demographic traits. Two of the key questions analyzed in this article are whether respondents “support the use of warning labels on misleading or false information?” and on “information that is potentially dangerous.” This section considers these two key questions in terms of combinations of demographic traits, as some intersection groups may have distinct characteristics. Statistical significance calculations (in the form of confidence intervals) for this data and analysis are included in Appendix B. The five intersections considered are combinations of age and political party affiliation, education level and political party affiliation, age and gender, education and gender, and political party affiliation and gender. Each intersection is considered for both warning label support questions.

**Figure 37:**
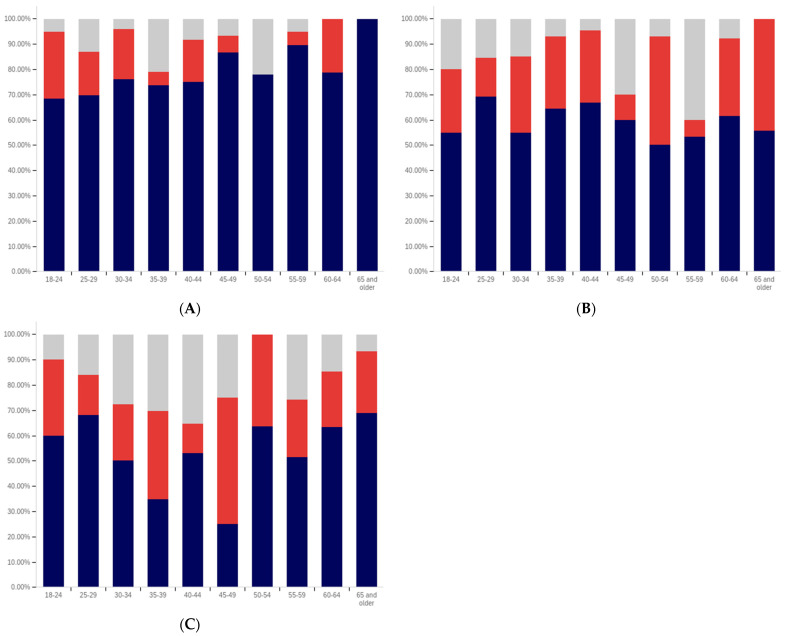
Should social media and news websites place warning labels on potentially misleading or false information?—by age and political party affiliation: (**A**) Democratic party (top left), (**B**) Republican Party (top right), and (**C**) independent/other party (bottom).

**Figure 38:**
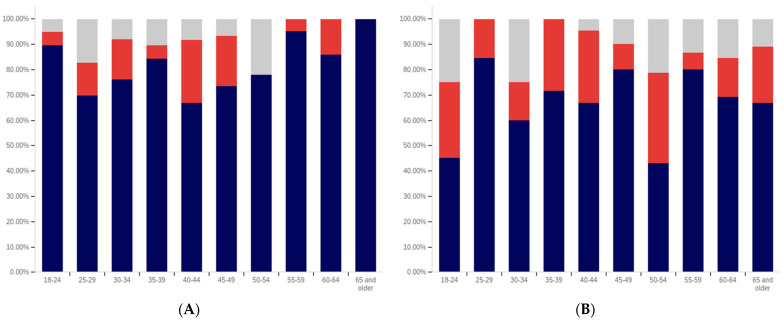

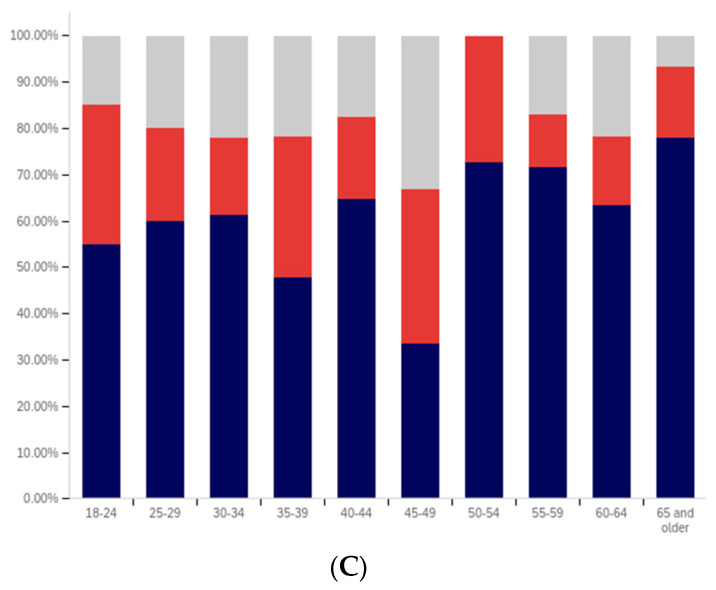
Should social media and news websites place warning labels on information that is potentially dangerous to your health?—by age and political party affiliation: (**A**) Democratic party (top left), (**B**) Republican Party (top right), and (**C**) independent/other party (bottom).

The intersection of the age and party affiliation demographics are analyzed in Figure 37 and Figure 38. As would be expected from the data analyzing party affiliation only, democrats have the highest level of support for warning labels, in all age groups, with approximately 70% or more of all respondents supporting labeling. Republicans have lower levels of support; however, in all cases approximately 50% or more of respondents support labeling. In most categories, there are a significant number of individuals opposed to labeling; however, this is less than the number of individuals supporting labeling. In all but two age groups (45–49 and 55–59), there are far more individuals opposed to labeling than uncertain (however, in these two groups, there is minimal opposition and far more confusion).

Independents show far more variability with regards to support for labeling. The 45–49 age group has only approximately 25% support for labeling; however, the 25–29, 50–54, 60–65 and 65+ age groups have approximately 65% to 70% support for labeling. There is also significant variability between opposition and confusion, with 50–54 having only supporting and opposed respondents and a minimal number of individuals indicting confusion in the 65+ age group. A general trend of the older and younger age groups supporting and the middle-range age groups opposing warning labeling is present.

The next question is similar, except dealing with information that is medically harmful as opposed to simply potentially misleading or false. While the general trend has been for greater support for labeling of medically harmful information, this does not hold for all groups under the Republicans and independents/other party supporters. Democrats show increases in many categories for warning labeling medically harmful information, though small declines are present in the 40–49 age demographic groups.

For Republicans, across all age levels, there are generally fewer respondents indicating uncertainty. However, support drops notably among 18–24-year-olds and 50–54-year-olds, while increasing in other demographic groups such as 25–29-year-olds, 30–34-year-olds, 45–49-year-olds and the 55+ age groups.

Independents show increases in support for warning labeling for medically harmful information in the 30+ age demographics and moderate declines in the 18–29 age demographics. A number of notable increases in support are present, such as in the 30–39 age demographics and the 55–59 demographic.

It is interesting that age groups are not performing harmoniously across the three political parties. The youngest group of respondents, 18–24-year-olds, shows declines for Republicans and independent/other party respondents, while support amongst democrats in this age group increases. The presence of several examples like this illustrates that the age-party affiliation intersection may be a valuable research analysis area, when seeking to understand data trends.

**Figure 39:**
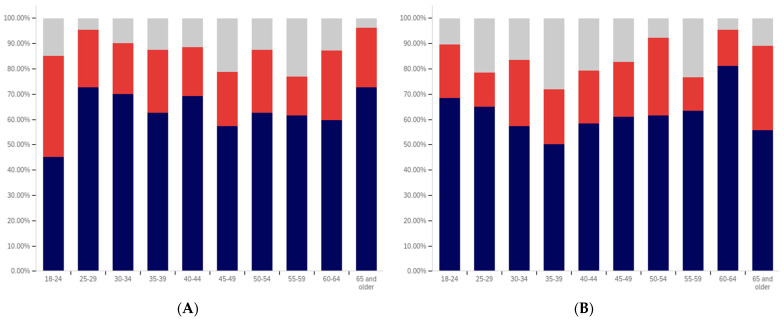
Should social media and news websites place warning labels on potentially misleading or false information?—by age and gender: (**A**) male (left), (**B**) female (right).

**Figure 40:**
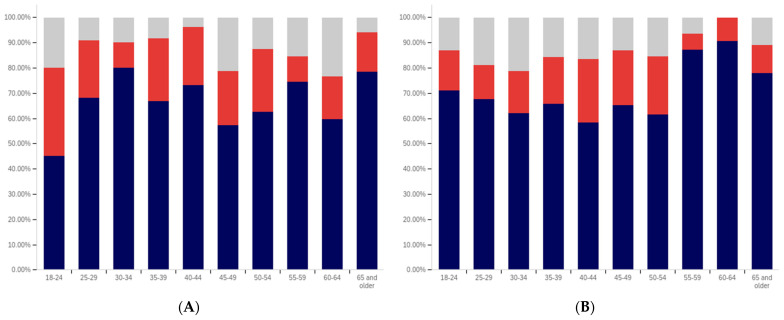
Should social media and news websites place warning labels on information that is potentially dangerous to your health?—by age and gender: (**A**) male (left), (**B**) female (right).

Focus now turns to comparing the support for warning labels for potentially misleading or false content between genders, across the different age groups. This data is shown in Figure 39 and Figure 40. Notably, the previous gender comparison indicated that both genders were relatively close in their perceptions of warning labels and support. However, when looking at the data in terms of the intersection of age and gender, a number of interesting patterns are present.

First, there is dramatically less support for warning labels amongst 18–24-year-old males than females. While males’ uncertainty is higher (approximately 15% versus 10%), only approximately 45% of 18–24-year-old males support warning labeling as opposed to approximately 70% of 18–24 year-old females. However, the next five age groups (25- to 49-year-olds) have higher male support for labeling, as compared to female support levels. The 50–59-year-old demographics have approximately the same level of support by both genders. Male 60–64-year-olds have notably less support for labeling (60% as opposed to 80% of respondents, with more uncertainty). Males over 65, on the other hand, support labeling in 70% of responses, as opposed to 55% (with females having slightly over double the reported uncertainty, albeit a small uncertainty value as compared to other age groups).

A similar trend is present with the support for medically dangerous warning labeling. Only 45% of 18–24-year-old males support the labeling, as compared to 70% of females in this age group. Notably, a number of less pronounced differences are also present at other age levels. Significantly higher support for warning labeling, by males, exists at the 30–34 and 40–44-year-old age levels. The 45–49 and 55–64-year-old males indicate less pronouncedly lower support levels. Similar levels of support are present at 25–29 years, 35–39 years, 50–54 years, and in the 65+ demographic.

**Figure 41:**
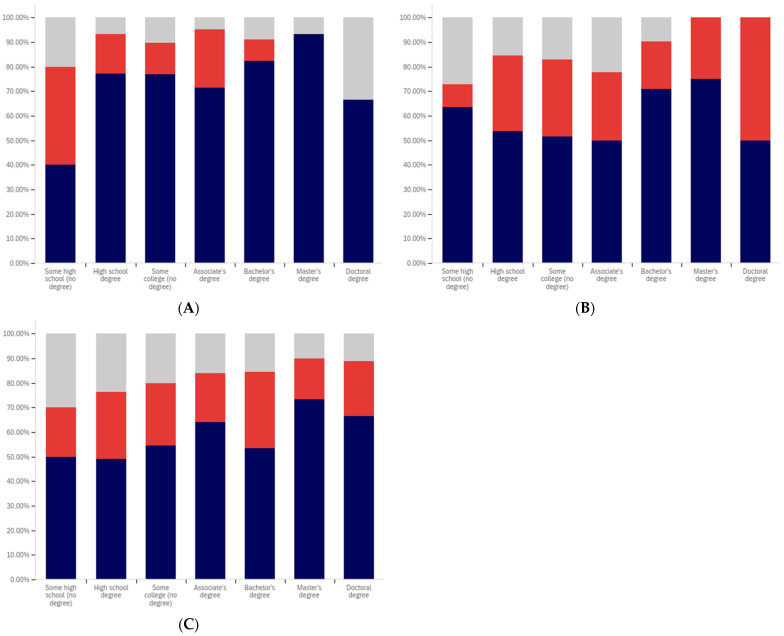
Should social media and news websites place warning labels on potentially misleading or false information?—by education and political party affiliation: (**A**) Democratic party (top left), (**B**) Republican Party (top right), and (**C**) independent/other party (bottom).

**Figure 42:**
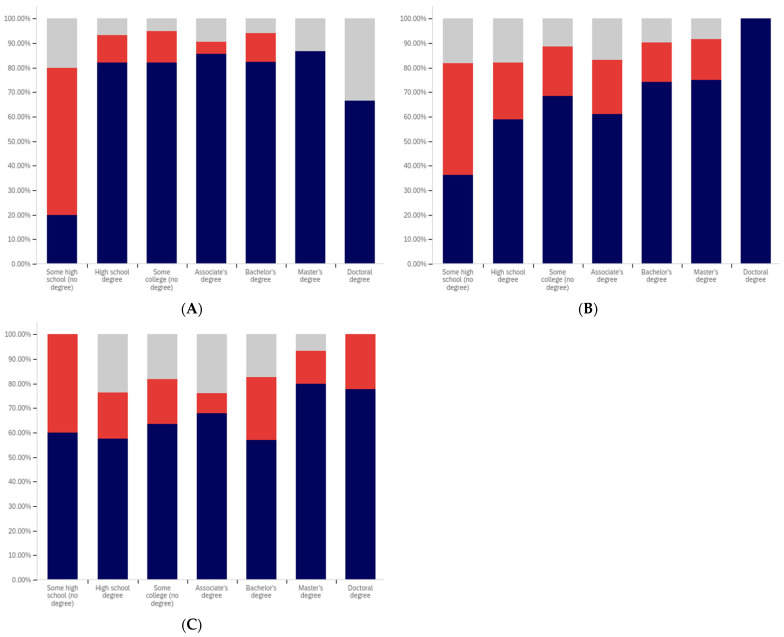
Should social media and news websites place warning labels on information that is potentially dangerous to your health?—by education level and political party affiliation: (**A**) Democratic party (top left), (**B**) Republican Party (top right) and (**C**) independent/other party (bottom).

Now, focus turns to analyzing the intersection of education and political party affiliation. This data is presented in Figure 41 and Figure 42.

A general trend of support for warning labeling increasing with educational attainment is present in both the Democrat and independent/other party respondents’ responses. In both cases, a slight dip in support (more notable with the Democrats) is present at the doctoral level. The Republican respondents, on the other hand, show a general trend of declining support for labeling with increasing education, through the associates degree level. Bachelor’s and master’s degree holders, however, show increase support as opposed to “no college” and “some college” respondents. Notably, there are insufficient respondents in the doctoral level Republican group to facilitate meaningful analysis at this educational level for Republicans.

Perhaps the most interesting portion of this data is the dramatic change that is noted between “some high school” and high school degree and higher-level educated democrats. Only 40% of “some high school” democrats indicate support for warning labeling. However, for those who have completed high school, support jumps to approximately 80%. Whether this is caused by the high school education or correlates with another factor is undetermined. Nevertheless, this is an interesting finding.

Like has been the case with the analysis of several other demographic characteristics, the results for the health-dangerous warning label support mirror, but show more pronounced trends, as compared to the warning label support for potentially misleading and false information. This data is presented in Figure 42.

The notably higher level of labeling support amongst Democrats (as compared to both independent/other party respondents and Republicans) is apparent across almost every educational level (note that the Republican doctorate holders are excluded from analysis due to the very small number of respondents in this category). The principal exception to this trend is the “some high school” educational level where only 20% of Democrats support warning labeling, as opposed to approximately 35% of Republicans (and 60% of independent/other party respondents). Interestingly, both the Republicans and the independent/other party respondents seem to increase in support for warning labeling with advancing levels of education. Democrats (for the high school degree, some college, associate’s degree, and master’s degree categories) show a relatively consistent level of support. This support drops somewhat with doctorate holders. It is also notable that, while there are approximately the same level of labeling supporters at the “some high school” level (versus high school graduates), for independents, all of the non-supporters of labeling are opposed to it (there are no uncertain responses), so the overall level of support is less pronounced (given that some uncertain individuals would likely go in both directions, if forced to choose, if they were present).

**Figure 43:**
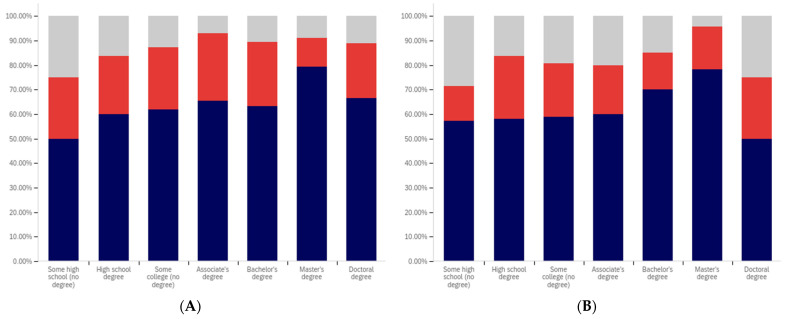
Should social media and news websites place warning labels on potentially misleading or false information?—by education and gender: (**A**) male (left), (**B**) female (right).

**Figure 44:**
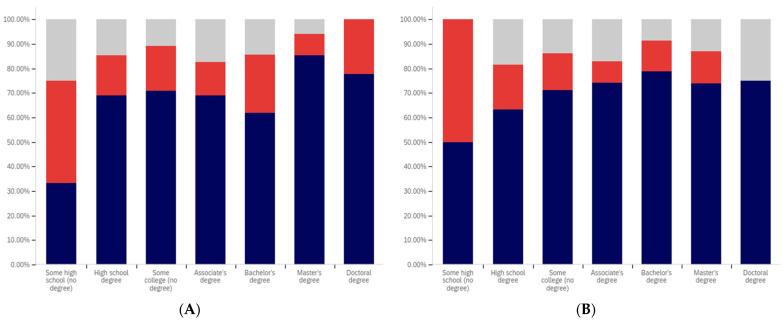
Should social media and news websites place warning labels on information that is potentially dangerous to your health?—by education and gender: (**A**) male (left), (**B**) female (right).

Focus now turns to analyzing the level of support for warning labeling based on the intersection of educational level and gender. This data is presented in Figure 43 and Figure 44. Largely, males and females have similar trends present across different educational levels. Fewer males indicated support at the “some high school” level, as compared to females. Females also show notably greater support at the bachelor’s degree level and notably less support than males at the doctoral degree holder level. These differences are relatively small, as compared to some of the more pronounced differences discussed previously in other analysis.

As has been the case with analysis under other demographic conditions, the patterns under the health-harmful warning labeling are more pronounced. This data is shown in Figure 44. Only 30% of “some high school” males and 50% of “some high school” females support labeling. Males have approximately 70% support in the high school graduates, “some college”, and associate’s categories. Females have similar support in the “some college” and associate’s degree categories, but only just over 60% of female high school graduates support health-harmful information warning labeling. A greater percentage of female respondents indicated warning labeling support at the bachelor’s, master’s, and doctoral levels, as compared to males.

**Figure 45:**
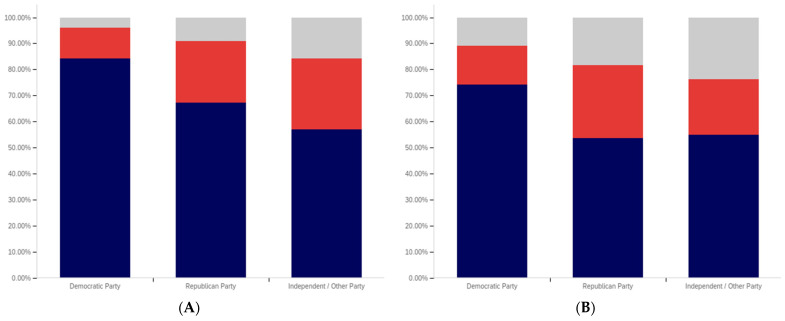
Should social media and news websites place warning labels on potentially misleading or false information?—by political party affiliation and gender: (**A**) male (left), (**B**) female (right).

**Figure 46:**
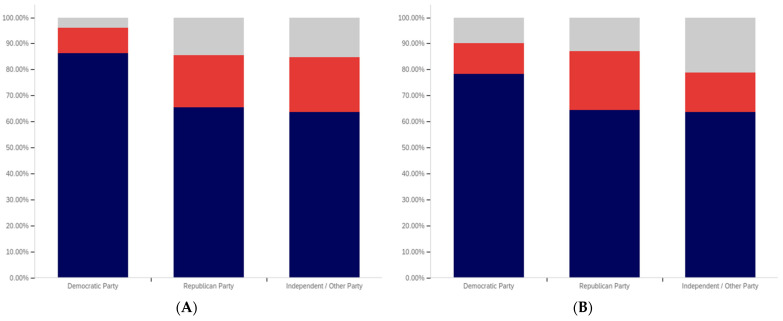
Should social media and news websites place warning labels on information that is potentially dangerous to your health?—by political party affiliation and gender: (**A**) male (left), (**B**) female (right).

Finally, focus turns to analyzing the intersection of political party affiliation and gender. This data is presented in Figure 45 and Figure 46. Comparing the data between males and females for the potentially misleading and false information labeling, greater support is present from males for labeling in each political party. Approximately 85% of Democrat males support labeling, as opposed to 75% of Democrat females. Approximately 70% of Republican males support labeling, as opposed to 55% of Republican females. The difference is less pronounced among independent/other party respondents, where just slightly more males than females support labeling. However, in the independent/other party respondents, there are more males opposed to labeling than females, while females have a greater level of uncertain respondents, as opposed to males.

With the medically harmful information labeling, for which data is shown in Figure 46, there is again a pronouncedly higher level of support amongst male Democrats as opposed to female ones. Male support is only marginally higher amongst Republicans and independent/other party respondents.

The foregoing has demonstrated that several intersection groups have pronouncedly different levels of support for labeling, as compared to analysis that considers only one group and not the intersection. In particular, amongst young, ‘some high school’ Democrats, there is a pronouncedly different view on labeling as compared to other Democrats. This far more negative view towards warning labeling is demonstrably different, also, from most other demographic intersection groups and the previously discussed non-intersecting demographic analysis.

## 11. Conclusions and Future Work

This paper has analyzed the potential value and perceptions of the use of, and support for online media content warning labeling. It has shown that the majority of Americans (75%) support the use of online media content warning labeling of potentially misleading or false information. Further, this study has shown that the level of support is, generally, even higher (80%) for labeling of medically harmful content. Despite these high levels of support, only 50% of respondents indicated self-efficacy of labeling, though higher levels of perceived efficacy were reported for others.

A number of notable demographically correlated findings were also present in the data. A clear correlation between age and warning label efficacy and support was shown to exist. This suggests that the efficacy of labeling will likely change over time and that label beliefs may be an impact of different eras of socialization. Youth and seniors showed the most awareness, with the former likely being due to general familiarity with social media, while the later may be attributable to senior-targeted awareness campaigns. Limited trends in education-related data were present, showing that the aforementioned age-related trends are not primarily due to education level correlation. Greater educational level attainment, in particular, was shown to reduce uncertainty. Additionally, a notable level of distrust was shown for dangerous-to-health labeling amongst the ‘some high school’ respondents. There were also not a lot of notable general trends with income level, though uncertainty was shown to decrease with greater income. Self-efficacy of labels was also shown to increase with income. Perhaps most interestingly, the percentage of respondents indicating that their own posts had been labeled declined with income level.

Gender-correlations presented some interesting findings. Males generally reported greater self-efficacy of labeling and perceived efficacy for others, though both genders reported a similar level of seeing post warning labels. Additionally, despite reporting greater self-efficacy, males indicated slightly less support for labeling. Finally, political party correlation analysis indicated greater Democrat efficacy and support for posts and greater labeling of Democrats’ posts (possibly due to greater awareness and willingness to label posts by other Democrats).

The demographic group intersection analysis data also presented a number of interesting findings. Age and gender intersection analysis indicated a notable difference in warning label support between males and females in the 18–24-year-old age group for both general labeling and medical harm-related labeling, with males reporting considerably less support than females. This is interesting as males (in general) reported more support than females overall. Males also reported higher levels of support in both the Democrat and Republican gender/political affiliation intersection analysis data, for the general warning labeling question; however, only Democrat males reported greater support for the medial harm-related question. Male and female support was generally similar across different age groups; however, there was a notable difference between male and female support for medical harm-related labeling in the 18–24-year-old age group, with only 30% of males supporting this labeling as opposed to 50% of females.

The age and political affiliation intersection analysis showed a much greater variance between different age levels of independents and other affiliations, than either Republicans or Democrats, suggesting that there may be differences in the precise beliefs and affiliations of this group across age groups. There was also notably less support by 18–24 and 50–54-year-old republicans when asked about medically harmful warning labeling (as compared to Democrats who also actually had higher support for this labeling in the 18–24 year-old age group).

The education level and political affiliation intersection data also yielded several interesting findings, though most areas were comparatively similar. Notably, the “some high school” Democrats had dramatically different labeling beliefs than other Democrats, with only half as much labeling support for the general question and a quarter as much support for the medical harm-related question. The Republican “some high school” respondents also showed a lower level of support for the medical harm-related question, but not for labeling in general.

Thus, while content warning labeling is generally supported by Americans, this study has shown that this support is not equal amongst all demographic groups. Some groups show greater levels of support for warning labeling than others. In a limited number of cases, groups were shown to be against content warning labeling. Demographics such as Democrats who have not completed high school, males who have not completed high school and 18–24-year-old males all had significantly more respondents indicating opposition to labeling, as compared to the population at large and significantly less respondents indicating support, as compared to the population.

It is also notable that respondents indicated a belief in the efficacy of warning labeling for others with greater frequency, as compared to its efficacy for themselves. This may mean that respondents are underestimating warning labels’ efficacy on themselves or overestimating its efficacy for others.

The tremendous power wielded by social media presents a clear and immediate need for techniques to mitigate the spread of intentionally deceptive online content. Warning labels may facilitate doing this without impairing free speech or the principles of democracy, personal liberty, and free enterprise. The public support shown by this survey suggests that Americans, in general, see labeling as an acceptable solution. Some groups, though, such as those described above may need additional outreach. This may, for some, be needed to improve their digital literacy. Others may benefit from a demonstration that labels won’t infringe on their personal freedoms.

The public support for warning labeling suggests that additional investigation is needed to ascertain—and perhaps seek ways to increase—their effectiveness. Given the key result of this study, that the population seems willing to have content labeled—if it is shown to be effective, labeling may represent a significant solution to the problems of intentionally deceptive and manipulative online content. The evaluation of the efficacy of labeling for this purpose is, thus, a key area of needed and planned future work.

Given this, one key area of additional analysis will be to ascertain whether all members of different demographic groups perceive warning labels in the same way. It is possible that the concept may have different meanings to some respondents in various demographic categories (for example, by age). Assessment of the perception of different types of warning labels, in general and by demographic groups, is a key area of future inquiry.

Other areas of potential future work include additional analysis of the data discussed herein and other related data to review associations between responses on various combinations of non-demographic questions. This would facilitate potentially identifying relationships between past occurrences and beliefs in one area and beliefs in other areas.

## Figures and Tables

**Table 1:** Respondents’ age distribution.

18–24	25–29	30–34	35–39	40–44	45–49	50–54	55–59	60–64	65 and Older
10.57%	10.93%	11.29%	10.04%	8.96%	6.63%	6.09%	12.54%	12.19%	10.75%
59	61	63	56	50	37	34	70	68	60

**Table 2:** Respondents’ education distribution.

Some High School (No Degree)	High School Degree	Some College (No Degree)	Associate’s Degree	Bachelor’s Degree	Master’s Degree	Doctoral Degree
4.68%	25.72%	23.20%	11.51%	22.12%	10.25%	2.52%
26	143	129	64	123	57	14

**Table 3:** Respondents’ household income level distribution.

$24,999 or Less	$25,000 to $49,999	$50,000 to $74,999	$75,000 to $99,999	$100,000 to $124,999	$125,000 or More
21.68%	19.18%	27.42%	16.13%	6.63%	8.96%
121	107	153	90	37	50

**Table 4:** Respondents’ political party identification distribution.

Democratic Party	Republican Party	Independent/Other Party
29.03%	26.70%	44.27%
162	149	247

**Table 5:** Respondents’ gender distribution.

Male	Female	Non-Binary
48.57%	50.72%	0.72%
271	283	4

**Table 6:** Nationally Representative Sample Characteristics.

Age	Gender	Household Income	Political Affiliation
18–34 33%	Female 50.5%	$0–$50k 40%	Democrat: 28%
35–55 32%	Male 49.5%	$50K–<$75K 28%	Republican: 27%
55+ 35%		$75K–<$100K 17%	Other: 45%
		$100K+ 15%	

## Data Availability

A data release, via a data journal publication, is planned once initial analysis of all data is complete.

## Appendix A. Confidence Intervals for Sections 4–9

This section presents confidence interval data (at a 95% confidence level) for Section 4, Section 5, Section 6, Section 7, Section 8 and Section 9 in Table A1, Table A2, Table A3, Table A4, Table A5, Table A6, Table A7, Table A8, Table A9, Table A10, Table A11 and Table A12.

**Table A1:** Confidence intervals for Section 4.

	Value	Interval
Figure 1	57.3%	53.0–61.7%
Figure 2	39.2%	34.9–43.4%
Figure 3	54.6%	50.2–59.0%
Figure 4	14.6%	11.5–17.7%
Figure 5	65.1%	60.9–69.3%
Figure 6	70.4%	66.4–74.4%

**Table A2:** Confidence intervals for Section 5, part 1.

	18–24		25–29		30–34	
	Value	Interval	Value	Interval	Value	Interval
Figure 7	72.2%	60.3–84.2%	75.5%	63.9–87.1%	63.2%	50.6–75.7%
Figure 8	38.9%	25.9–51.9%	45.3%	31.9–58.7%	43.1%	30.4–55.8%
Figure 9	52.8%	39.4–66.3%	52.8%	39.4–66.3%	56.9%	44.2–69.6%
Figure 10	14.8%	5.3–24.3%	17.0%	6.9–27.1%	22.4%	11.7–33.1%
Figure 11	61.1%	48.1–74.1%	69.8%	57.5–82.2%	65.5%	53.3–77.7%
Figure 12	64.8%	52.1–77.6%	69.8%	57.5–82.2%	70.7%	59.0–82.4%

**Table A3:** Confidence intervals for Section 5, part 2.

	35–39		40–44		45–49	
	Value	Interval	Value	Interval	Value	Interval
Figure 7	56.0%	42.2–69.8%	58.7%	44.5–72.9%	54.5%	37.6–71.5%
Figure 8	32.0%	19.1–44.9%	43.5%	29.2–57.8%	33.3%	17.2–49.4%
Figure 9	54.0%	40.2–67.8%	52.2%	37.7–66.6%	45.5%	28.5–62.4%
Figure 10	16.0%	5.8–26.2%	23.9%	11.6–36.2%	12.1%	1.0–23.3%
Figure 11	58.0%	44.3–71.7%	69.6%	56.3–82.9%	57.6%	40.7–74.4%
Figure 12	66.0%	52.9–79.1%	71.7%	58.7–84.8%	60.6%	43.9–77.3%

**Table A4:** Confidence intervals for Section 5, part 3.

	50–54		55–59		60–64	
	Value	Interval	Value	Interval	Value	Interval
Figure 7	54.8%	37.3–72.4%	51.6%	39.3–63.8%	51.7%	38.9–64.6%
Figure 8	25.8%	10.4–41.2%	42.2%	30.1–54.3%	35.1%	22.7–47.5%
Figure 9	38.7%	21.6–55.9%	61.9%	49.9–73.9%	55.2%	42.4–68.0%
Figure 10	16.1%	3.2–29.1%	6.3%	0.3–12.2%	13.8%	4.9–22.7%
Figure 11	61.3%	44.1–78.4%	61.9%	49.9–73.9%	69.0%	57.1–80.9%
Figure 12	61.3%	44.1–78.4%	81.3%	71.7–90.8%	70.7%	59.0–82.4%

**Table A5:** Confidence intervals for Section 5, part 4.

	65+	
	Value	Interval
Figure 7	34.0%	21.2–46.7%
Figure 8	44.2%	30.7–57.7%
Figure 9	64.2%	51.2–77.1%
Figure 10	5.7%	0.0–11.9%
Figure 11	73.6%	61.7–85.5%
Figure 12	77.4%	66.1–88.6%

**Table A6:** Confidence intervals for Section 6, part 1.

	Some High School		High School Degree		Some College	
	Value	Interval	Value	Interval	Value	Interval
Figure 13	36.8%	15.2–58.5%	52.1%	43.2–61.0%	66.4%	57.8–75.0%
Figure 14	21.1%	2.7–39.4%	33.9%	25.5–42.3%	38.8%	29.9–47.7%
Figure 15	50.0%	26.9–73.1%	53.3%	44.4–62.3%	60.7%	51.8–69.5%
Figure 16	10.5%	−3.3–24.3%	14.0%	7.9–20.2%	17.9%	11.0–24.9%
Figure 17	52.6%	30.2–75.1%	60.8%	52.1–69.6%	62.4%	53.6–71.2%
Figure 18	36.8%	15.2–58.5%	66.1%	57.7–74.5%	73.5%	65.5–81.5%

**Table A7:** Confidence intervals for Section 6, part 2.

	Associate’s Degree		Bachelor’s Degree		Master’s Degree	
	Value	Interval	Value	Interval	Value	Interval
Figure 13	56.9%	44.2–69.6%	59.8%	50.9–68.7%	53.7%	40.4–67.0%
Figure 14	36.2%	23.8–48.6%	41.9%	32.9–50.8%	56.6%	43.3–69.9%
Figure 15	41.4%	28.7–54.1%	53.8%	44.8–62.9%	64.8%	52.1–77.6%
Figure 16	20.7%	10.3–31.1%	12.0%	6.1–17.8%	9.3%	1.5–17.0%
Figure 17	63.8%	51.4–76.2%	66.7%	58.1–75.2%	81.5%	71.1–91.8%
Figure 18	72.4%	60.9–83.9%	69.2%	60.9–77.6%	83.3%	73.4–93.3%

**Table A8:** Confidence intervals for Section 6, part 3.

	Doctoral Degree	
	Value	Interval
Figure 13	50.0%	21.7–78.3%
Figure 14	33.3%	6.7–60.0%
Figure 15	50.0%	21.7–78.3%
Figure 16	16.7%	0.0–37.8%
Figure 17	66.7%	40.0–93.3%
Figure 18	75.0%	50.5–99.5%

**Table A9:** Confidence intervals for Section 7, part 1.

	$24,999 or Less		$25,000 to $49,999		$50,000 to $74,999	
	Value	Interval	Value	Interval	Value	Interval
Figure 19	55.3%	45.7–64.9%	54.2%	44.2–64.1%	58.3%	50.1–66.5%
Figure 20	29.1%	20.4–37.9%	38.5%	28.8–48.3%	41.7%	33.5–49.9%
Figure 21	48.5%	38.9–58.2%	54.2%	44.2–64.1%	57.2%	49.0–65.5%
Figure 22	15.4%	8.5–22.3%	18.8%	10.9–26.6%	15.1%	9.2–21.1%
Figure 23	62.1%	52.8–71.5%	57.3%	47.4–67.2%	69.8%	62.2–77.4%
Figure 24	65.4%	56.2–74.5%	68.8%	59.5–78.0%	71.9%	64.5–79.4%

**Table A10:** Confidence intervals for Section 7, part 2.

	$75,000 to $99,999		$100,000 to $124,999		$125,000 or More	
	Value	Interval	Value	Interval	Value	Interval
Figure 19	54.9%	44.1–65.6%	69.7%	54.0–85.4%	60.9%	46.8–75.0%
Figure 20	43.2%	32.4–54.0%	39.4%	22.7–56.1%	47.8%	33.4–62.3%
Figure 21	56.1%	45.4–66.8%	60.6%	43.9–77.3%	54.3%	40.0–68.7%
Figure 22	13.4%	6.0–20.8%	12.1%	1.0–23.3%	6.5%	−0.6–13.7%
Figure 23	69.5%	59.5–79.5%	66.7%	50.6–82.8%	65.2%	51.5–79.0%
Figure 24	73.2%	63.6–82.8%	69.7%	54.0–85.4%	76.1%	63.8–88.4%

**Table A11:** Confidence intervals for Section 8.

	Democrat		Republican		Independent	
	Value	Interval	Value	Interval	Value	Interval
Figure 25	62.7%	54.7–70.6%	58.1%	49.8–66.4%	53.4%	46.8–60.0%
Figure 26	50.0%	41.8–58.2%	31.4%	23.6–39.2%	37.0%	30.6–43.4%
Figure 27	58.9%	50.7–67.0%	54.4%	46.0–62.8%	52.0%	45.4–58.6%
Figure 28	19.7%	13.2–26.3%	14.6%	8.7–20.5%	11.3%	7.1–15.5%
Figure 29	79.4%	72.8–86.1%	59.9%	51.6–68.1%	59.3%	52.8–65.8%
Figure 30	82.4%	76.1–88.7%	65.7%	57.7–73.6%	65.6%	59.3–71.9%

**Table A12:** Confidence intervals for Section 9.

	Male		Female	
	Value	Interval	Value	Interval
Figure 31	58.1%	52.0–64.3%	56.4%	50.3–62.5%
Figure 32	42.9%	36.7–49.1%	35.6%	29.7–41.5%
Figure 33	56.6%	50.3–62.8%	53.4%	47.2–59.6%
Figure 34	14.6%	10.2–19.1%	14.3%	10.0–18.7%
Figure 35	65.0%	59.1–71.0%	64.8%	58.9–70.7%
Figure 36	69.1%	63.3–74.9%	71.7%	66.1–77.3%

## Appendix B. Confidence Intervals for Section 10

This section presents confidence interval data (at a 95% confidence level) for Section 10 in Table A13, Table A14, Table A15, Table A16, Table A17, Table A18, Table A19, Table A20, Table A21 and Table A22.

**Table A13:** Confidence intervals for Figure 37 in Section 10.

	Democrat		Republican		Independent	
	Value	Interval	Value	Interval	Value	Interval
18–24	70.6%	48.9–92.2%	52.6%	30.2–75.1%	61.1%	38.6–83.6%
25–29	73.7%	53.9–93.5%	69.2%	44.1–94.3%	66.7%	46.5–86.8%
30–34	75.0%	57.7–92.3%	61.1%	38.6–83.6%	56.3%	31.9–80.6%
35–39	76.5%	56.3–96.6%	61.5%	35.1–88.0%	40.0%	18.5–61.5%
40–44	81.8%	59.0–100.0%	73.7%	53.9–93.5%	56.3%	31.9–80.6%
45–49	91.7%	76.0–100.0%	55.6%	23.1–88.0%	25.0%	0.5–49.5%
50–54	75.0%	45.0–100.0%	50.0%	23.8–76.2%	66.7%	35.9–97.5%
55–59	87.5%	71.3–100.0%	50.0%	23.8–76.2%	54.5%	37.6–71.5%
60–64	81.8%	59.0–100.0%	60.0%	29.6–90.4%	67.6%	52.5–82.7%
65+	100.0%	100.0–100.0%	62.5%	29.0–96.0%	71.8%	57.7–85.9%

**Table A14:** Confidence intervals for Figure 38 in Section 10.

	Democrat		Republican		Independent	
	Value	Interval	Value	Interval	Value	Interval
18–24	100.0%	100.0–100.0%	47.4%	24.9–69.8%	50.0%	26.9–73.1%
25–29	68.4%	47.5–89.3%	84.6%	65.0–100.0%	61.9%	41.1–82.7%
30–34	75.0%	57.7–92.3%	66.7%	44.9–88.4%	68.8%	46.0–91.5%
35–39	82.4%	64.2–100.0%	69.2%	44.1–94.3%	50.0%	28.1–71.9%
40–44	72.7%	46.4–99.0%	73.7%	53.9–93.5%	68.8%	46.0–91.5%
45–49	75.0%	50.5–99.5%	77.8%	50.6–100.0%	33.3%	6.7–60.0%
50–54	75.0%	45.0–100.0%	42.9%	16.9–68.8%	77.8%	50.6–100.0%
55–59	94.1%	82.9–100.0%	78.6%	57.1–100.0%	75.8%	61.1–90.4%
60–64	90.9%	73.9–100.0%	60.0%	29.6–90.4%	67.6%	52.5–82.7%
65+	100.0%	100.0–100.0%	62.5%	29.0–96.0%	76.9%	63.7–90.1%

**Table A15:** Confidence intervals for Figure 39 in Section 10.

	Male		Female	
	Value	Interval	Value	Interval
18–24	47.4%	24.9–69.8%	67.6%	51.9–83.4%
25–29	68.4%	47.5–89.3%	69.7%	54.0–85.4%
30–34	70.0%	49.9–90.1%	62.2%	46.5–77.8%
35–39	63.6%	43.5–83.7%	53.6%	35.1–72.0%
40–44	72.0%	54.4–89.6%	66.7%	46.5–86.8%
45–49	57.1%	31.2–83.1%	57.9%	35.7–80.1%
50–54	50.0%	10.0–90.0%	64.0%	45.2–82.8%
55–59	62.9%	46.8–78.9%	60.7%	42.6–78.8%
60–64	61.0%	46.0–75.9%	88.2%	72.9–100.0%
65+	75.6%	63.0–88.1%	62.5%	29.0–96.0%

**Table A16:** Confidence intervals for Figure 40 in Section 10.

	Male		Female	
	Value	Interval	Value	Interval
18–24	47.4%	24.9–69.8%	73.5%	58.7–88.4%
25–29	68.4%	47.5–89.3%	69.7%	54.0–85.4%
30–34	80.0%	62.5–97.5%	67.6%	52.5–82.7%
35–39	68.2%	48.7–87.6%	64.3%	46.5–82.0%
40–44	76.0%	59.3–92.7%	66.7%	46.5–86.8%
45–49	57.1%	31.2–83.1%	63.2%	41.5–84.8%
50–54	50.0%	10.0–90.0%	64.0%	45.2–82.8%
55–59	77.1%	63.2–91.1%	86.2%	73.7–98.8%
60–64	61.0%	46.0–75.9%	94.1%	82.9–100.0%
65+	77.8%	65.6–89.9%	75.0%	45.0–100.0%

**Table A17:** Confidence intervals for Figure 41 in Section 10.

	Democrat		Republican		Independent	
	Value	Interval	Value	Interval	Value	Interval
Some high school	50.0%	1.0–99.0%	62.5%	29.0–96.0%	42.9%	6.2–79.5%
High school degree	79.4%	65.8–93.0%	51.4%	35.2–67.5%	55.1%	41.2–69.0%
Some college	77.1%	63.2–91.1%	52.9%	36.2–69.7%	58.3%	44.4–72.3%
Associate’s degree	72.2%	51.5–92.9%	53.3%	28.1–78.6%	64.0%	45.2–82.8%
Bachelor’s degree	81.8%	68.7–95.0%	70.0%	53.6–86.4%	55.6%	42.3–68.8%
Master’s degree	100.0%	100.0–100.0%	81.8%	59.0–100.0%	72.4%	56.1–88.7%
Doctoral degree	66.7%	13.3–100.0%	100.0%	100.0–100.0%	62.5%	29.0–96.0%

**Table A18:** Confidence intervals for Figure 42 in Section 10.

	Democrat		Republican		Independent	
	Value	Interval	Value	Interval	Value	Interval
Some high school	25.0%	0.0–67.4%	25.0%	0.0–55.0%	57.1%	20.5–93.8%
High school degree	82.9%	70.4–95.3%	56.8%	40.8–72.7%	61.2%	47.6–74.9%
Some college	85.7%	74.1–97.3%	70.6%	55.3–85.9%	66.7%	53.3–80.0%
Associate’s degree	83.3%	66.1–100.0%	66.7%	42.8–90.5%	68.0%	49.7–86.3%
Bachelor’s degree	81.8%	68.7–95.0%	73.3%	57.5–89.2%	59.3%	46.2–72.4%
Master’s degree	92.9%	79.4–100.0%	81.8%	59.0–100.0%	79.3%	64.6–94.1%
Doctoral degree	66.7%	13.3–100.0%	100.0%	100.0–100.0%	75.0%	45.0–100.0%

**Table A19:** Confidence intervals for Figure 43 in Section 10.

	Male		Female	
	Value	Interval	Value	Interval
Some high school	37.5%	4.0–71.0%	63.6%	35.2–92.1%
High school degree	60.0%	45.7–74.3%	60.8%	49.7–71.9%
Some college	65.3%	52.0–78.6%	59.7%	48.0–71.4%
Associate’s degree	64.3%	46.5–82.0%	63.3%	46.1–80.6%
Bachelor’s degree	64.4%	53.4–75.4%	70.5%	57.0–83.9%
Master’s degree	78.8%	64.8–92.7%	85.7%	70.7–100.0%
Doctoral degree	66.7%	35.9–97.5%	66.7%	13.3–100.0%

**Table A20:** Confidence intervals for Figure 44 in Section 10.

	Male		Female	
	Value	Interval	Value	Interval
Some high school	25.0%	0.0–55.0%	45.5%	16.0–74.9%
High school degree	68.9%	55.4–82.4%	64.0%	53.1–74.9%
Some college	73.5%	61.1–85.8%	74.6%	64.2–85.0%
Associate’s degree	67.9%	50.6–85.2%	76.7%	61.5–91.8%
Bachelor’s degree	63.0%	51.9–74.1%	79.5%	67.6–91.5%
Master’s degree	84.8%	72.6–97.1%	81.0%	64.2–97.7%
Doctoral degree	77.8%	50.6–100.0%	66.7%	13.3–100.0%

**Table A21:** Confidence intervals for Figure 45 in Section 10.

	Male		Female	
	Value	Interval	Value	Interval
Democrat	84.4%	73.9–95.0%	76.8%	68.4–85.3%
Republican	66.0%	53.3–78.8%	55.4%	44.7–66.1%
Independent	58.8%	50.9–66.7%	59.7%	48.4–71.1%

**Table A22:** Confidence intervals for Figure 46 in Section 10.

	Male		Female	
	Value	Interval	Value	Interval
Democrat	86.7%	76.7–96.6%	80.2%	72.2–88.2%
Republican	64.2%	51.2–77.1%	66.3%	56.1–76.4%
Independent	65.5%	57.9–73.2%	66.7%	55.8–77.6%

## References

[1] Jahng M.R. (2021). Is Fake News the New Social Media Crisis? Examining the Public Evaluation of Crisis Management for Corporate Organizations Targeted in Fake News. Int. J. Strateg. Commun..

[2] Hopf H., Krief A., Mehta G., Matlin S.A. (2019). Fake science and the knowledge crisis: Ignorance can be fatal. R. Soc. Open Sci..

[3] Sellnow T.L., Parrish A., Semenas L. (2019). From Hoax as Crisis to Crisis as Hoax: Fake News and Information Disorder as Disruptions to the Discourse of Renewal. J. Int. Crisis Risk Commun. Res..

[4] Lazer D.M.J., Baum M.A., Benkler Y., Berinsky A.J., Greenhill K.M., Menczer F., Metzger M.J., Nyhan B., Pennycook G., Rothschild D. (2018). The science of fake news. Science.

[5] Chen E., Chang H., Rao A., Lerman K., Cowan G., Ferrara E. (2021). COVID-19 misinformation and the 2020 U.S. presidential election. Harv. Kennedy Sch. Misinf. Rev..

[6] Gillin J. How Pizzagate Went from Fake News to a Real Problem for a D.C. Business. PolitiFact 2016. https://www.politifact.com/article/2016/dec/05/how-pizzagate-went-fake-news-real-problem-dc-busin/.

[7] Aisch G., Huang J., Kang C. (2016). Dissecting the #PizzaGate Conspiracy Theories—The New York Times. New York Times.

[8] Roozenbeek J., Schneider C.R., Dryhurst S., Kerr J., Freeman A.L.J., Recchia G., Van Der Bles A.M., Van Der Linden S. (2020). Susceptibility to misinformation about COVID-19 around the world. R. Soc. Open Sci..

[9] Gupta L., Gasparyan A.Y., Misra D.P., Agarwal V., Zimba O., Yessirkepov M. (2020). Information and Misinformation on COVID-19: A Cross-Sectional Survey Study. J. Korean Med. Sci..

[10] Hern A. (2020). Twitter to remove harmful fake news about coronavirus. Guardian.

[11] Kien G. (2020). Postmodernism Trumps All: The World Without Facts. Qual. Inq..

[12] Koschorke A., Thomas M., Rossman S. (2019). Facts Shifting to the Left: From Postmodernism to the Postfactual Age. PMLA.

[13] Roth Y., Pickles N. Updating Our Approach to Misleading Information. https://web.archive.org/web/20210806095419/https://blog.twitter.com/en_us/topics/product/2020/updating-our-approach-to-misleading-information.

[14] Facebook How Is Facebook Addressing False Information through Independent Fact-Checkers?. https://www.facebook.com/help/1952307158131536.

[15] Samek G. Greater Transparency for Users around News Broadcasters. https://blog.youtube/news-and-events/greater-transparency-for-users-around/.

[16] Lyons K., Lawler R. A New Facebook Whistleblower Has Come Forward with More Allegations—The Verge. https://www.theverge.com/2021/10/22/22741024/facebook-new-whistleblower-allegations-sec.

[17] Ziady H. Facebook Kept Oversight Board in the Dark about Its “Cross-Check” Program. https://www.cnn.com/2021/10/21/tech/facebook-cross-check-oversight-board/index.html.

[18] Zadrozny B. “Carol’s Journey”: What Facebook Knew about How It Radicalized Users. https://www.nbcnews.com/tech/tech-news/facebook-knew-radicalized-users-rcna3581.

[19] Sicha C. The New Facebook Name: What’s Next?. https://nymag.com/intelligencer/2021/10/facebooks-name-change-horizon.html.

[20] Tartarus. https://www.britannica.com/topic/Tartarus.

[21] Shao C., Ciampaglia G.L., Varol O., Flammini A., Menczer F. (2017). The spread of fake news by social bots. arXiv.

[22] Borchers A.T., Hagie F., Keen C.L., Gershwin M.E. (2007). The history and contemporary challenges of the US food and drug administration. Clin. Ther..

[23] Huizinga M.M., Carlisle A.J., Cavanaugh K.L., Davis D.L., Gregory R.P., Schlundt D.G., Rothman R.L. (2009). Literacy, Numeracy, and Portion-Size Estimation Skills. Am. J. Prev. Med..

[24] US Food and Drug Administration Changes to the Nutrition Facts Label. https://www.fda.gov/food/food-labeling-nutrition/changes-nutrition-facts-label.

[25] Spradling M., Straub J., Strong J. (2021). Protection from ‘Fake News’: The Need for Descriptive Factual Labeling for Online Content. Futur. Internet.

[26] Motion Picture Association Inc., National Association of Theatre Owners Inc. (2020). Classification and Rating Rules.

[27] WELCOME TO FilmRatings.com. https://www.filmratings.com/.

[28] The V-Chip: Options to Restrict What Your Children Watch on TV|Federal Communications Commission. https://www.fcc.gov/consumers/guides/v-chip-putting-restrictions-what-your-children-watch.

[29] Ratings—ESRB Ratings. https://www.esrb.org/ratings/.

[30] Ratings Process—ESRB Ratings. https://www.esrb.org/ratings/ratings-process/.

[31] U.S. Government (1791). The First Amendment to the United States Constitution.

[32] Akin H., Yeo S.K., Wirz C.D., Scheufele D.A., Brossard D., Xenos M.A., Corley E.A. (2018). Are attitudes toward labeling nano products linked to attitudes toward GMO? Exploring a potential ‘spillover’ effect for attitudes toward controversial technologies. J. Responsible Innov..

[33] Wong A.W.-T. (2019). Analysis of Global Regulatory Schemes on Chance-Based Microtransactions. Asper Rev. Int. Bus. Trade Law.

[34] Borin N., Cerf D.C., Krishnan R. (2011). Consumer effects of environmental impact in product labeling. J. Consum. Mark..

[35] Baade B. (2018). Fake News and International Law. Eur. J. Int. Law.

[36] Ott B. (2005). Some Good News about the News: 5 Reasons Why ‘Fake’ News is Better than Fox ‘News’—Flow. Flow.

[37] Saez-Trumper D. Fake Tweet Buster: A Webtool to Identify Users Promoting Fake News on Twitter. Proceedings of the HT’14.

[38] Conroy N.J., Rubin V.L., Chen Y. Automatic Deception Detection: Methods for Finding Fake News. Proceedings of the ASIST.

[39] Peters J.W. (2016). Wielding Claims of ‘Fake News,’ Conservatives Take Aim at Mainstream Media. New York Times.

[40] European Commission Online Disinformation. https://digital-strategy.ec.europa.eu/en/policies/online-disinformation.

[41] United Nations Educational, Scientific and Cultural Organization Journalism, “Fake News” and Disinformation: A Handbook for Journalism Education and Training. https://en.unesco.org/fightfakenews.

[42] Gelfert A. (2018). Fake News: A Definition. © Axel Gelfert. Informal Log..

[43] Jackson R.R., Cross F.R., Fiona Cross C.R., Le Comber S. (2013). A cognitive perspective on aggressive mimicry. J. Zool..

[44] Tornero J.M.M., Tayie S.S., Tejedor S., Pulido C. (2018). How to confront fake news through news literacy? State of the art. Doxa Comun..

[45] Kucharski A. (2016). Post-truth: Study epidemiology of fake news. Nature.

[46] Barua Z., Barua S., Aktar S., Kabir N., Li M. (2020). Effects of misinformation on COVID-19 individual responses and recommendations for resilience of disastrous consequences of misinformation. Prog. Disaster Sci..

[47] Scheibenzuber C., Hofer S., Nistor N. (2021). Designing for fake news literacy training: A problem-based undergraduate online-course. Comput. Human Behav..

[48] Bonnet J.L., Rosenbaum J.E. (2019). “Fake news,” misinformation, and political bias: Teaching news literacy in the 21st century. Commun. Teach..

[49] Grace L., Hone B. Factitious: Large scale computer game to fight fake news and improve news literacy. Proceedings of the Extended Abstracts of the 2019 CHI Conference on Human Factors in Computing Systems.

[50] Marchi R. (2012). With Facebook, Blogs, and Fake News, Teens Reject Journalistic “Objectivity”. J. Commun. Inq..

[51] Shearer E., Matsa K.E. News Use across Social Media Platforms 2018. https://www.pewresearch.org/journalism/2018/09/10/news-use-across-social-media-platforms-2018/.

[52] Fatilua J. (2018). Who trusts social media?. Comput. Human Behav..

[53] Literat I., Chang Y.K., Hsu S.Y. (2020). Gamifying fake news: Engaging youth in the participatory design of news literacy games. Converg. Int. J. Res. New Media Technol..

[54] Miller L.M.S., Cassady D.L. (2012). Making healthy food choices using nutrition facts panels. The roles of knowledge, motivation, dietary modifications goals, and age. Appetite.

[55] Bettman J.R., Payne J.W., Staelin R. (2018). Cognitive Considerations in Designing Effective Labels for Presenting Risk Information. J. Public Policy Mark..

[56] McAuliffe R. (2018). The FTC and the Effectiveness of Cigarette Advertising Regulations. J. Public Policy Mark..

[57] Asam E.H., Bucklin L.P. (2018). Nutrition Labeling for Canned Goods: A Study of Consumer Response. J. Mark..

[58] Andrews J.C., Netemeyer R.G., Durvasula S. (2018). Believability and Attitudes toward Alcohol Warning Label Information: The Role of Persuasive Communications Theory. J. Public Policy Mark..

[59] Gao M., Xiao Z., Karahalios K., Fu W.T. (2018). To label or not to label: The effect of stance and credibility labels on readers’ selection and perception of news articles. Proc. ACM Hum. -Comput. Interact..

[60] Pennycook G., Cannon T.D., Rand D.G. (2018). Prior exposure increases perceived accuracy of fake news. J. Exp. Psychol. Gen..

[61] Tangherlini T.R., Shahsavari S., Shahbazi B., Ebrahimzadeh E., Roychowdhury V. (2020). An automated pipeline for the discovery of conspiracy and conspiracy theory narrative frameworks: Bridgegate, Pizzagate and storytelling on the web. PLoS ONE.

[62] Duke University When It Comes to Fake News, People Desiring Chaos Are Undeterred by Warnings on Potential Misinformation. https://phys.org/news/2021-11-fake-news-people-desiring-chaos.html.

[63] Ardèvol-Abreu A., Delponti P., Rodríguez-Wangüemert C. (2020). Intentional or inadvertent fake news sharing? Fact-checking warnings and users’ interaction with social media content. Prof. Inf..

[64] Wright S.A. (1991). Reconceptualizing Cult Coercion and Withdrawal: A Comparative Analysis of Divorce and Apostasy. Soc. Forces.

[65] Kim A., Moravec P., Dennis A.R. (2019). When Do Details Matter? Source Rating Summaries and Details in the Fight against Fake News on Social Media. Source Rating Summaries and Details in the Fight against Fake News on Social Media (September 6, 2019).

[66] Fuhr N., Giachanou A., Grefenstette G., Gurevych I., Hanselowski A., Jarvelin K., Jones R., Liu Y., Mothe J., Nejdl W. (2018). An Information Nutritional Label for Online Documents. ACM SIGIR Forum.

[67] Vincentius K., Aggarwal P., Sahan A., Högden B., Madan N., Bangaru A., Schwenger C., Muradov F., Aker A. (2018). Information Nutrition Labels: A Plugin for Online News Evaluation. Proceedings of the First Workshop on Fact Extraction and VERification.

[68] Suttle R., Hogan S., Aumaugher R., Spradling M., Merrigan Z., Straub J. (2021). University Community Members’ Perceptions of Labels for Online Media. Futur. Internet.

[69] Khairunissa K. (2020). University Students’ Ability in Evaluating Fake News on Social Media. Rec. Libr. J..

[70] Coleman K. Introducing Birdwatch, a Community-Based Approach to Misinformation. https://blog.twitter.com/en_us/topics/product/2021/introducing-birdwatch-a-community-based-approach-to-misinformation.

[71] Meta Meta Journalism Project. https://www.facebook.com/journalismproject/.

[72] Qualtrics International I. Online Panels: Get Responses for Surveys & Research. https://www.qualtrics.com/research-services/online-sample/.

[73] (2010). AAPOR Opt-In Online Panel Task Force Research Synthesis: AAPOR Report on Online Panels. Public Opin. Q..

[74] U.S. Census Bureau American Community Survey Data. https://www.census.gov/programs-surveys/acs/data.html.

[75] Gallup Corporation Party Affiliation. https://news.gallup.com/poll/15370/party-affiliation.aspx.

[76] Brenan M. Biden’s Approval Rating Hits New Low of 43%. Harris’ Is 49%. https://news.gallup.com/poll/354872/biden-approval-rating-hits-new-low-harris.aspx.

[77] Ward M. Why Biden’s Poll Numbers Are Dropping. https://www.politico.com/newsletters/politico-nightly/2021/10/20/why-bidens-poll-numbers-are-dropping-494772.

[78] Gallup Congress and the Public | Gallup Historical Trends. https://news.gallup.com/poll/1600/congress-public.aspx.

[79] Overwhelming Majority of Americans Support Criminal Justice Reform, New Poll Finds|Vera Institute. https://www.vera.org/blog/overwhelming-majority-of-americans-support-criminal-justice-reform-new-poll-finds.

[80] Blizzard R. National Poll Results. https://www.politico.com/f/?id=00000161-2ccc-da2c-a963-efff82be0001.

[81] Long C., Fingerhut H. AP-NORC Poll: Nearly All in US Back Criminal Justice Reform. https://apnews.com/article/police-us-news-ap-top-news-politics-kevin-richardson-ffaa4bc564afcf4a90b02f455d8fdf03.

[82] Auxier B., Anderson M. (2021). Social Media Use in 2021. Pew Res. Cent..

[83] Marquit M., Schmidt J. What Is The Rule Of 55?. https://www.forbes.com/advisor/retirement/rule-of-55-retirement/.

[84] McDonald’s Restaurants Senior Discount. https://www.seniordiscounts.com/FeaturedDiscounts/McDonalds.aspx.

[85] 2021 Biggest List of Senior Discounts (Restaurants, Retail, Travel & More). https://www.theseniorlist.com/senior-discounts/.

[86] Gabriel M.G., Brown A., León M., Outley C. (2020). Power and Social Control of Youth during the COVID-19 Pandemic. Leis. Sci.

[87] Courtney D., Watson P., Battaglia M., Mulsant B.H., Szatmari P. (2020). COVID-19 Impacts on Child and Youth Anxiety and Depression: Challenges and Opportunities. Can. J. Psychiatry.

